# The Influence of COVID-19 Pandemic on Management Earnings Forecasts

**DOI:** 10.3389/fpsyg.2022.918560

**Published:** 2022-07-01

**Authors:** Xiangfei Fu, Yuanze Xu, Fangfang Zhou, Libin Zhao

**Affiliations:** ^1^School of Accounting, Institute of Intelligent Management Accounting and Internal Control, Nanjing Audit University, Nanjing, China; ^2^School of Economics and Management, Beijing Jiaotong University, Beijing, China; ^3^School of Accounting, Wuhan Textile University, Wuhan, China

**Keywords:** the COVID-19 pandemic, management earnings forecasts, media monitoring, legal environment, on-site monitoring activities

## Abstract

As the biggest black swan event of 2020, the COVID-19 pandemic has significantly weakened the ability of corporate stakeholders to monitor companies on site. In this context, exploring whether the on-site supervision restrictions triggered by the COVID-19 pandemic affect management earnings forecast disclosure is crucial to protect investors' interests and promote the stable development of the capital market. Based on quarterly data of Chinese A-share listed companies' earnings forecasts, this paper finds that: First, when the company's registry region is more severely affected by the COVID-19 pandemic, the company has less willingness to disclose its management earnings forecast. And those released forecasts tend to have lower qualities. Second, a higher level of media monitoring and a better legal environment can mitigate the negative impacts of the COVID-19 pandemic on both the willingness and the quality of management earnings forecast disclosure. Furthermore, mediating effect analysis shows that, the reduced on-site monitoring activities that were originally implemented by independent directors, institutional investors, and analysts during the epidemic period greatly limited stakeholders' monitoring efficiency, and thus cause significant influence on the disclosure of management earnings forecasts.

## Introduction

In 2020, the COVID-19 epidemic swept through the country like a flood. After the outbreak of the pandemic, the Chinese government took resolute and effective measures to control and to further prevent the spread of the epidemic, such as prohibiting personnel movement and requesting home quarantine. In contrast, the international anti-epidemic situation is not optimistic. The new coronavirus brought by people from abroad and overseas cold-chain cargoes has impeded the deployment of anti-epidemic measures in Beijing, Shanghai, Tianjin, and many other places. Predictably, the adverse effects of COVID-19 will remain for a long time. One obvious effect caused by the COVID-19 pandemic is that the large-scale movement of people will be strictly restricted and thus the ability of stakeholders to monitor companies will be greatly impeded, adding more uncertainties to the unfavorable situation of economic downturn overlaid with the impact of the epidemic, as well as threatening the protection of investors' interests and the stability of the capital market. This article focuses on exploring whether the COVID-19 pandemic will affect management earnings forecast disclosure due to reduced on-site supervision activities, and further offers suggestions to mitigate the adverse impact of the COVID-19 based on empirical evidence.

A management earnings forecast is a key way for investors to get essential information on corporate earnings. Current studies mainly focused on regional characteristics, company characteristics, and executive characteristics, and their influences on management earnings forecast disclosure, leaving the potential impact of major public emergency events untested. Therefore, in the context of the global spread of COVID-19, exploring the connection between major public emergencies and management earnings forecasts, as well as the potential mechanism of the stakeholders' on-site monitoring abilities in the pandemic era will help regulators and listed companies improve their response mechanisms and preventive measures, thereby protecting the core interests of investors and ensuring the smooth development of the capital market.

In this article, we argue that driven by opportunistic motives, management manipulates not only the timing or amount of earnings forecast disclosure but also the precision of earnings forecast disclosure. Stakeholders such as independent directors, analysts, and institutional investors have the motivation and ability to monitor managers and limit their opportunistic behaviors (Elyasiani et al., 2010). On-site monitoring activities allow stakeholders such as independent directors, analysts, and institutional investors to obtain “soft information” through “face-to-face” communication and site visits to the company's operation, production, and R&D activities (Jiang and Yuan, 2018). However, in the wake of the COVID-19 pandemic, stakeholders have either proactively reduced their personnel movements for fear of infection or because of government travel restrictions.

Based on the major public emergency of the COVID-19 outbreak, this article examines the impact of the on-site monitoring restrictions on the disclosure of management earnings forecasts. We find that: companies tend to provide fewer and less accurate voluntary management earnings forecasts when their headquarters are highly affected by the pandemic; and a higher level of media monitoring, as well as a better legal environment can effectively mitigate the negative impact of COVID-19 on management earnings forecast disclosure. The mechanism test indicates that it is common for companies to choose online board meetings instead of offering opportunities to institutional investors and analysts to implement site visits. This effect will greatly reduce the ability of independent directors to monitor companies on site, which in turn affects the disclosure of management earnings forecasts.

The contributions of this paper are mainly in the following areas: First, this paper focuses on the economic consequences of COVID-19 by taking the management earnings forecast as the landing point, which helps to expand the study of the economic consequences of COVID-19 from the macro and meso levels to the corporate governance level. Studies have focused on the impact of the COVID-19 on macro-level economic development (Walmsley et al., 2021) and international trade (Chen and Mao, 2020), and the only studies that have been conducted on companies have focused only on the negative impact of COVID-19 on their operations (Eggers, 2020).

Second, this study takes the COVID-19 as an entry point to explore the impact of major unexpected public events on the disclosure of management performance forecasts and enriches the research related to the impact factors of management earnings forecasts disclosure from the perspective of public event shocks. Studies have predominantly focused on regional characteristics (Baginski et al., 2002), firm characteristics (Francis et al., 2008), and executive characteristics (Brockman et al., 2010) for the disclosure of management earnings forecasts, and few of them discussed the impact of public emergencies. This article empirically examines the mechanism and the effect of COVID-19 on the disclosure of management earnings forecasts, which helps to enrich the research related to the factors influencing the disclosure of management earnings forecasts.

Third, combining the analysis of media monitoring and the legal environment, this paper provides appropriate countermeasures to deal with the adverse effects on corporate governance practices caused by the COVID-19 pandemic. We find that external governance factors such as media monitoring and the legal environment can mitigate the negative effects of COVID-19 on the disclosure of management earnings forecasts, and provide policy recommendations on how to mitigate the impact of COVID-19.

The remainder of this study is organized as follows. In Section Related Literature and Hypothesis Development, we review studies and develop the main hypotheses. Section Research Design introduces the research design and the sample. Section Control Variable presents the empirical results and conducts cross-sectional tests. In Section Mechanism Test, we report the mechanism test results. Finally, Section Robustness Test concludes the study.

## Related Literature and Hypothesis Development

### Economic Consequences of COVID-19

As a major public health emergency, the contagious and dangerous nature of COVID-19 has a serious impact on the economic operating system. Currently, studies on the economic consequences of COVID-19 have focused on three main areas: the impact of COVID-19 on economic development, international trade, and business operations.

In terms of COVID-19's impact on economic development, Walmsley et al. (2021) illustrate that COVID-19 has caused the US GDP to decline by 20.3% and the employment ratio to decline by 22.4%from February to April 2020, compared with the same period in the last year. Using Google search volume as a proxy variable for investor attention, Smales (2021) finds that the COVID-19 affects investor attention and investment sentiment, which in turn cause a negative impact on financial market stability.

Regarding international trade, Cao et al. (2020) and Chen and Mao (2020) find that the COVID-19 has a significant impact on agriculture, which leads to a gradual shrinkage of agricultural trade. A study by Vidya and Prabheesh (2020) notes a sharp decline in trade links between countries under the impact of COVID-19. Fu (2020) argues that COVID-19 affects international trade and global value chains through three paths: cutting off the logistics supply chain, disrupting production supply, and reducing consumer demand.

About company operations, Eggers (2020) conclude that the outbreak of COVID-19 has devastated small, medium, and micro enterprises (MSMEs) that lack external resources to protect themselves. Hassan et al. (2020) and Kerr (2020) find that COVID-19 has plunged companies into a crisis of declining orders and supply chain disruptions. De Vito and Gomez (2020) simulated the cash use of firms with operational constraints and found that the firm with partial operational flexibility depletes its cash stock in about 2 years after the outbreak of COVID-19.

In summary, previous studies have focused on the impact of COVID-19 on economic development and international trade at the macro level, and these studies have focused on the negative impact of COVID-19 on firm operations, with less attention paid to the economic consequences of COVID-19 on micro-level corporate governance.

### Factors Influencing the Disclosure of Management Earnings Forecasts

Compared with other sources of accounting information, management earnings forecasts enable investors to be informed of corporate surplus information in advance and help reduce the degree of information asymmetry in the capital market, thus becoming the focus of attention in theoretical and practical fields. Currently, studies on the factors influencing the disclosure of management earnings forecasts have focused on three areas: regional characteristics, company features, and executive characteristics.

In terms of the impact of regional characteristics on the disclosure of management earnings forecasts, Baginski et al. (2002) show that the deterrent effect of external litigation risk reinforces managers' willingness and quality of earnings forecasts when the regional legal system is well established. Johnson et al. (2001), using a sample of high-tech companies, also find that the regional rule of the legal environment can increase the frequency of management earnings forecasts.

Regarding the company characteristics, some studies suggest that when firms have a strong demand for external financing, there is a greater incentive to reduce information asymmetry with investors and lower financing costs through high levels of earnings forecasts (Francis et al., 2008). Based on the product market competition perspective, Li (2010) finds that competition from potential entrants motivates firms to increase the frequency of earnings forecasts, while competitors will decrease. In addition, it has been shown that less performance earnings are disclosed when the company is under a higher level of uncertainty (Bozanic et al., 2018) and with higher financial risk (McNichols, 1989), while when the shareholdings of institutional investors in the company are lower (Ajinkya et al., 2005) and the board size is smaller (Karamanou and Vafeas, 2005), the progress of earnings forecasts will be lower.

Regarding the executive characteristics of management earnings forecasts, it has been found that private interest motives such as executive option exercise (Brockman et al., 2010) and insider stock trading (Cheng et al., 2013) can significantly affect management earnings forecast. In addition, when the level of executive overconfidence is higher, the accuracy of management earnings forecasts is lower (Hribar and Yang, 2016).

In summary, existing studies have focused on the impact of regional characteristics, company features, and executive characteristics on the disclosure of management earnings forecasts, and less on the impact of significant public emergencies.

### Research Hypotheses

Management earnings forecasts provide the capital market with private information about the company's operations and help to mitigate the degree of information asymmetry with investors, thus reducing the cost of capital and litigation risk for companies (Hirst et al., 2008). However, the current system of earnings forecasts in China is semi-compulsory, and managers have greater discretion as whether to issue an earnings announcement, as well as the content and the timing of the forecasts. The existence of agency problems makes managers reluctant to disclose earnings forecasts because it would enhance investor and shareholder scrutiny of management, thus limiting its scope for opportunistic behavior (Nagar et al., 2003). Cheng and Lo (2006) find that agency problems cause executives to increase the disclosure of negative earnings forecasts before buying shares to reduce stock prices. Ertimur et al. (2014) find that management delays disclosing negative earnings forecasts until large shareholders sell shares unlocked by the IPO. Driven by opportunistic motives, managers will manipulate not only the timing or amount of earnings forecast disclosure but also the accuracy (Rogers and Stocken, 2005; Billings and Buslepp, 2016). Cheng et al. (2013) find that executives tend to issue more accurate positive earnings forecasts and more ambiguous negative earnings forecasts to boost stock prices before selling their stocks. The above study shows that management opportunism can have a significant impact on earnings forecasts.

Stakeholders such as independent directors, analysts, and institutional investors have the motivation and ability to monitor management teams and limit their opportunistic behaviors (Elyasiani et al., 2010). On-site monitoring enables stakeholders to obtain “soft information” that cannot be simply stored and recorded through “face-to-face” communication and on-site visits to company operations, production and R&D activities, and to verify the authenticity of “hard information” from financial reports (Jiang and Yuan, 2018). However, in the wake of the COVID-19 pandemic, stakeholders like directors, analysts, and institutional investors have either proactively reduced their movements due to fears of viral infection or because of government restrictions. Directors are currently unable to attend meetings and discussions of listed companies at that time. Meanwhile, it is difficult for analysts and institutional investors to conduct field research. The above impacts will result in a significant reduction in the ability of stakeholders to monitor companies on-site, making it difficult to effectively curb opportunistic behaviors, which in turn will affect the disclosure of earnings forecasts. Accordingly, our first hypothesis is stated below:

H1: When headquarter cities are highly affected by COVID-19, companies are less likely to issue voluntary management earnings forecasts, and the accuracy of earnings forecasts is even lower.

Analysis in the previous section shows that the COVID-19 has significantly reduced the on-site monitoring ability of stakeholders, making it difficult to effectively curb managers' opportunistic behaviors, which consequently affects the disclosure of management earnings forecasts. Further exploration of the external governance factors that can mitigate the negative impact of the COVID-19 on management performance forecast disclosure is presented here. As an effective external governance mechanism, media monitoring contributes to restraining opportunistic behaviors from managers such as manipulation of surplus (Chahine et al., 2015). Miller (2006) and Dyck et al. (2010) demonstrate the role that media monitoring can play in curbing opportunistic management behaviors such as accounting fraud or extreme surplus management. Chen et al. (2021) find that the rise in the number of media reports can effectively curb the degree of accrual surplus management and real activity surplus management of firms, indicating that the media, as external monitoring, can curb managers' opportunistic surplus management. Consequently, management opportunistic behavior has been effectively curbed when the intensity of media regulation is relatively higher. At this point, the impact of the on-site supervision restrictions triggered by COVID-19 is not apparent. Conversely, when the intensity of media regulation is weak, the weakened external oversight enables management to have more sufficient opportunistic behaviors. In this case, the impact of on-site monitoring restrictions triggered by COVID-19 on managers' opportunistic behaviors, which consequently act on management earnings forecast disclosure, will be more significant.

Based on the above analysis, our second hypothesis is stated as follows:

H2: higher level of media monitoring activities could significantly reduce the negative impact of COVID-19 on management earnings forecast disclosure.

The above analysis explores the impact of media monitoring activities on managers' opportunistic behaviors and further illustrates the important role played by media monitoring as an external governance factor during the COVID-19 pandemic.

The legal environment can likewise play an equally vital role in external governance and can reduce the managers' manipulation of surpluses (Francis et al., 2016). Burgstahler et al. (2006) find that different degrees of legal system construction lead to significant differences in surplus management among listed companies in each country. As a result, it is more difficult for managers to engage in opportunistic behaviors when the legal environment in the company's registry region is well-developed. In this case, the on-site oversight restrictions arising from the COVID-19 outbreak cannot have a significant impact. Conversely, when the rule of the legal environment in the region is poor, the absence of external oversight mechanisms makes it easier for management to engage in opportunistic behaviors. At this moment, the extent to which the on-site monitoring restrictions triggered by the COVID-19 affect managers' opportunistic behaviors and further affect management earnings forecast disclosure will be more obvious. Based on the above analysis, our third hypothesis is stated as follows:

H3: A better legal environment can significantly reduce the negative impact of COVID-19 on the disclosure of management earnings forecasts.

**Figure 1:**
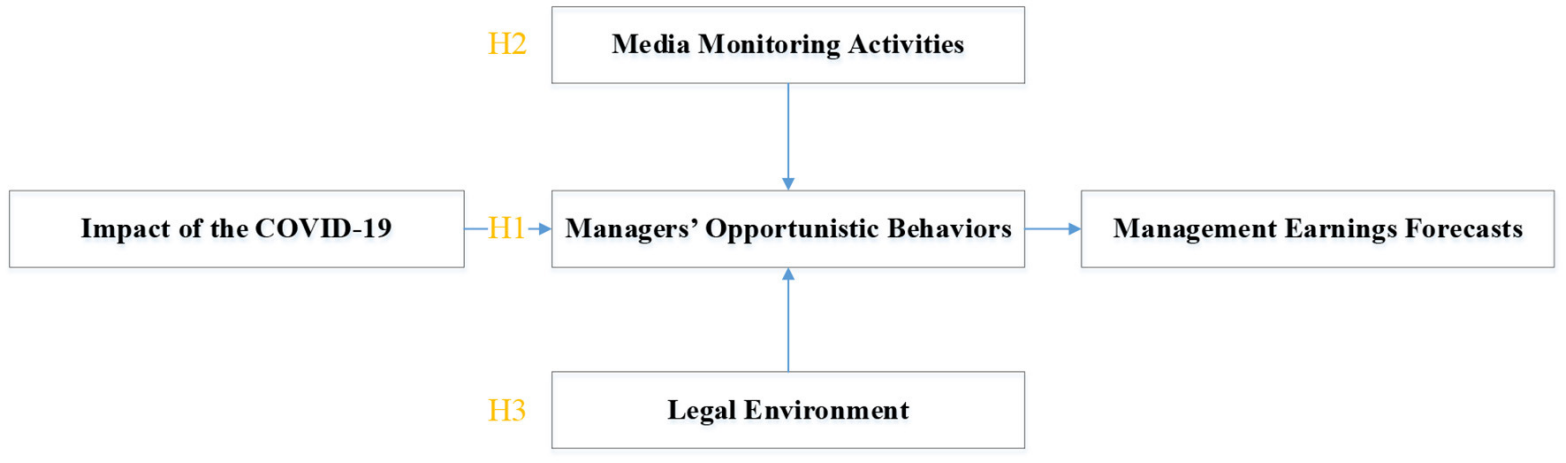
Theoretical logic.

As shown in Figure 1, H1: due to the influence of management opportunism, when headquarter cities are highly affected by COVID-19, companies are less likely to issue voluntary management earnings forecasts, and the accuracy of earnings forecasts is even lower. H2: listed companies with more media monitoring will limit the opportunistic behavior of management, so the negative impact of COVID-19 on the management's earnings forecast will be smaller. H3: listed companies with a better legal environment will limit the opportunistic behavior of management, so the negative impact of COVID-19 on the management's earnings forecast will be smaller. These three hypotheses aim to prove that COVID-19 affects management's earnings forecast by affecting management's opportunistic behavior.

## Research Design

### Data and Sample Selection

This article uses the quarterly data of earnings forecasts for the study of listed companies in 2020, which are treated as follows: (1) exclude the financial industry sample; (2) exclude the sample with incomplete data. Ultimately, 5,563 observations were obtained in this paper. To exclude the effect of outliers, this paper winsorized all continuous variables at 1 and 99%, and all regression standard errors were treated with the industry-level cluster method.

We extracted the management earnings forecasts data and the COVID-19 pandemic data from the CSMAR database. Data measuring the percentage of board meetings held online is obtained as follows: First, use Python's Selenium and Urllib library to obtain each listed company's board resolution announcement from Cninfo Network. Then, manually determine the number of board meetings corresponding to the announcement of board resolutions between the beginning of each year and the date of the financial report. Finally, we use Python's Pdfminer library to convert the downloaded PDF announcement into TXT format, select “communication” as the keyword for determining the form of a board meeting, conduct a keyword search on the above TXT text, determine whether the board meeting was held an online, and use it to calculate the percentage of board meetings held online. The response status of major public health emergencies in each province (autonomous region and municipality directly under the central government) was obtained from the official website of the Public Government of each province (autonomous region and municipality directly under the central government), and other data were obtained from the WIND database and CSMAR database.

### Variable Definitions

#### Dependent Variable

The explanatory variables in this study are management earnings forecasts, which are examined in terms of the voluntariness of management performance announcements and the accuracy of management earnings announcements, respectively.

##### Voluntary Forecasting of Management (Vol)

According to the information disclosure rules of the Shanghai and Shenzhen Stock Exchange, there are voluntary and mandatory earnings forecasts for listed companies in China. If a listed company suffers a first loss, turns a loss into a profit, or experiences substantial fluctuations in performance compared to the same period last year, it is mandatory to disclose earnings forecasts in a timely manner, except for a few cases where exemptions can be obtained, and the remaining cases can voluntarily disclose earnings forecasts. Therefore, if the type of earnings forecast issued by the company slightly increases, slightly decreases and there is a renewal of earnings, it is defined as voluntary earnings forecast, and Vol is set to 1, if it is another type, it is set to 0.

##### Accuracy of Management Earnings Forecast (Type)

Following Ajinkya et al. (2005), the accuracy of management earnings forecasts were classified into four grades from high to low: When management gives a clear forecast of future net profit, the variable Type takes the value of 4; if management gives a forecast of future net profit with both upper and lower limits, Type takes the value of 3; if it only gives an upper or lower limit of future net profit, Type takes the value of 2; if it gives only a qualitative description of future net profit without a clear value in the forecast, Type takes a value of 1.

#### Independent Variable

The explanatory variable in this paper is the impact of COVID-19, which is measured using two main measures.

##### Number of People Who Are Still in Treatment (Num)

The cumulative number of confirmed cases of newly diagnosed COVID-19 patients in the company's registry region as of the time of the earnings forecasts is measured by adding one to the natural logarithm of this number.

If the registry region of the company is more severely affected by the epidemic, the epidemic control measures to restrict the movement of people are more stringent. In this case, the more difficult it is for independent directors and other interested parties to go to the locally listed company for on-site supervision.

##### Level 1 Response Status (RHELevel-1)

The variable RHELevel-1 equals 1 if the province (autonomous region or municipality directly under the central government) where the listed company is located is in the first level response status of major public health emergencies as of the publication of the earnings forecast, and 0 if otherwise.

According to the “People's Republic of China Emergency Response Law”, “Prevention and Control of Infectious Diseases Law”, “National Public Health Emergency Response Plan”, “Public Health Emergency Response Regulations”, “Public Health Emergency Grading Standards”, based on the nature of the event and degree of harm, the scope of public health emergencies are divided into four levels, particularly significant (I), major (II), larger (III), and general (IV).

Traffic regulation measures are the most critical of all epidemic prevention measures and have the greatest inhibitory effects on the development of the epidemic. According to the above-mentioned laws and regulations, in the emergency response status of public health emergencies of special significance (level I), public governments at all levels can: delineate controlled areas, after reporting for approval to implement blockades of infectious disease areas; take compulsory measures to restrict or stop fairs, rallies, theater performances, and other crowd gathering activities, as well as suspend work, business, and school; management of mobile populations, implement preventive work, and control measures; implement transportation health quarantine, quarantine inspection, and other restrictive measures for people traveling to and from the area. Therefore, if the province (autonomous region or municipality directly under the Central Government) where the company is located is in the first level response status of major public health emergencies, the epidemic control measures to restrict the movement of people are more stringent.

#### Moderating Variables

##### Media Monitoring

The dummy variable takes the value of 1 if the number of media reports and online reports for listed companies is greater than the median and 0 if otherwise.

##### Legal Environment

A dummy variable that takes the value of 1 if the registry region of the company is located in four provinces (municipalities directly under the Central Government) of Guangdong Province, Shanghai, Zhejiang Province, and Jiangsu Province and 0 if otherwise (Wang et al., 2008).

This article refers to Cheng et al. (2011), which argue that regions with higher marketization process also have higher degrees of intermediary development, better legal system environments, stronger protection for small and medium-sized investors, and companies' misrepresentations are more likely to be detected by the market and regulatory authorities, so the legal environment of a region can be measured by its marketization process. In terms of variable definition, Cheng et al. (2011) regarded Guangdong province, Shanghai, Zhejiang Province, and Jiangsu Province, which ranked in the top 5 in the report on China's Provincial Marketization Index from 2004 to 2007, as regions with high marketization processes, and other provinces as regions with low marketization processes. As the latest research result of the National Economic Research Institute, the report on China's Provincial Marketization Index aims to evaluate the overall situation and progress in different aspects of the marketization reform of China's provinces, autonomous regions, and municipalities in the past period.

The method has been widely used in subsequent studies. Fan et al. (2011) point out that the marketization process is a comprehensive indicator of the external environment of companies, which is a series of changes in the economic, social, and legal system, while Li and Liu (2016) argue that the distinctive features of regions with low marketization processes are: unsound construction of legal regulations and inadequate regulation of information disclosure, and choose Fan and Wang's China Marketization Index-−2011 Annual Report on the Relative Process of Marketization by Regions sets four provinces, namely Guangdong, Shanghai, Zhejiang, and Jiangsu, as the high marketization process group. Zhu and Jia (2017) argue that in regions with high marketization processes, laws and regulations are relatively sound and the market competition mechanism is more complete, which can alleviate the financing constraints of enterprises to a certain extent, and use the marketization index of each region in “China Marketization Index-−2011 Annual Report on the Relative Process of Marketization by Regions” as a reference to measure the regional marketization process, taking the four provinces ranked in the top four for five consecutive years, Guangdong Province, Shanghai, Jiangsu Province, and Zhejiang Province, as regions with high marketisation process. Li et al. (2018) argue that regions with low marketization process will inhibit the enterprises' R&D behavior due to the lack of legal protection for R&D activities, and set the four provinces of Guangdong Province, Shanghai, Zhejiang Province, and Jiangsu Province, which were ranked in the top five for six consecutive years from 2004 to 2009 in “China Marketization Index-−2011 Annual Report on the Relative Process of Marketization by Regions”, as regions with a better institutional environment.

#### Control Variables

Following existing studies (Ajinkya et al., 2005; Drobetz et al., 2017), this article controls a set of variables that may affect management earnings forecasts. Publicly listed firms with better financial performance are more motivated to provide positive signals to the market and enhance voluntary disclosure; therefore, control variables characterizing firms' financial characteristics include firm size (Size), profitability (ROA), financial leverage (Lev), and growth (Growth). The better the internal and external governance mechanism of a firm and the more robust the monitoring mechanism for management, the more likely it is to disclose high-quality management performance forecasts. Therefore, the control variables characterizing the internal and external governance characteristics of a firm include equity concentration (Top1), the proportion of independent directors (Indep), board size (Board), dual positions (Dual), years of IPO (Age), nature of ownership (SOE), firm characteristics (Big4), institutional investor ownership (Institution), and analyst tracking (Analyst). In addition, the study by Ajinkya et al. (2005) finds that the higher the industry concentration, the higher the accuracy of the companies' earnings forecasts. Thus, Herfindahl Index (HHI) is also selected as the control variable. Finally, this article also controls for industry fixed effects. The definitions of the main variables involved in the empirical test in this paper are shown in Table 1.

**Table 1:** Variable definitions.

**Variable**	**Variable definition**
Vol	If the type of earnings forecast issued by the company is slightly increased, slightly decreased and renewed earnings, it is defined as voluntary earnings forecast and takes the value of 1, if other types take the value of 0.
Type	The point forecast takes the value of 4, the closed interval forecast takes the value of 3, the open interval forecast takes the value of 2, and the qualitative forecast takes the value of 1.
Num	Number of patients with in hospital in the listed company's local administrative district as of the time of the earnings announcement, adding one taking the natural logarithm.
RHELevel-1	As of the publication of the results forecast, the value is 1 if the listed company is located in a province (autonomous region or municipality directly under the Central Government) with a Level 1 response status for major public health emergencies, and 0 otherwise.
Media	The value is 1 if the number of media reports and online reports on the listed company is greater than the median, otherwise it is 0.
RLE	If the registry region of the listed company is in four provinces (municipalities directly under the Central Government) of Guangdong Province, Shanghai, Zhejiang Province and Jiangsu Province take the value of 1, otherwise it is 0.
Size	Natural logarithm of the company's total assets at the end of the year.
ROA	Net profit/average total assets.
Lev	Total liabilities at end of period/total assets at end of period.
Growth	Growth rate of main business revenue.
Top1	Percentage of shareholding of the largest shareholder.
Indep	Number of independent directors/total number of board of directors.
Board	Natural logarithm of the number of board members.
Dual	1 if the chairman and general manager are both appointed by the same person, 0 otherwise.
Age	Difference between sample year and company listing year.
SOE	Take 1 if it is a state-owned enterprise, otherwise 0.
Big4	1 if international “Big Four” accounting firm, 0 otherwise.
Institution	Institutional investors' shareholding ratio.
Analyst	Natural logarithm of the number of analysts who track and publish research reports.
HHI	Sum of the squares of the top five shareholders' shareholdings.
Industry	According to the industry standard of “Industry Classification Guidelines for Listed Companies” (2001 version) of China Securities Regulatory Commission, manufacturing industry is classified by secondary code and others are classified by primary code.

*This table provides the definitions of all variables*.

### Empirical Models

To test the hypothesis of this paper, a regression model (1) was constructed based on the Healy et al. (1992) model, and OLS regression was used to examine the impact of COVID-19 on the management's earnings forecasts, which in turn tested the H1 proposed in this paper.


(1) $$\begin{array}{l} EarningsForecast\;=\;\beta_{0}\;+\;\beta_{1}Num/RHELevel\;-\;1\\ +\;\beta_{2}Controls\;+\;IND\;+\;\varepsilon \end{array}$$

In Equation (1), Earnings forecast is the explanatory variable measuring management earnings forecasts, including voluntariness of earnings forecasts (Vol) and accuracy of earnings forecasts (Type); Num and RHELevel-1 are the explanatory variables of the degree of impact of COVID-19; controls are a series of control variables. In addition, this article controls industry fixed effects (Industry Effect) in model regression, and the industry classification criteria are based on the Guidelines for Industry Classification of Listed Companies (2012 Revision).

## Empirical Results

### Descriptive Statistics

Table 2 provides descriptive statistics for the main variables in this paper. As Table 2 shows, the mean value of the voluntary nature of earnings forecasts (Vol) is 0.2574, indicating that 25.74% of the sample voluntarily disclosed their earnings forecasts. The mean value of the forecast accuracy (Type) is 2.7235 and the median is 3, showing that the majority of the sample uses a closed interval forecast. From the perspective of the explanatory variables, the mean value of Num was 0.9891, reflecting a mean of 1.6888 (e^0.9891^-1) cumulative confirmed cases of the COVID-19 at the locality district as of the time of the earnings forecasts. The average of RHELevel-1 is 0.1738, indicating that 17.38% of the provinces (autonomous regions and municipalities directly under the Central Government) where the sample is located are in Level 1 response status for major public health emergencies as of the time of the earnings forecasts. As for the moderating variables, the mean value of media monitoring (Media) was 0.4774, indicating that 47.74% of the sample had a greater than the median number of media reports and online coverage. The average of Law Environment (RLE) was 0.4859, reflecting that 48.59% of the sample had registered in Guangdong Province, Shanghai, Zhejiang Province, and Jiangsu Province.

**Table 2:** Descriptive statistics.

**Variable**	***N***	**Mean**	**S.D**.	**Min**	**P25**	**Median**	**P75**	**Max**
Vol	5,563	0.2574	0.4372	0.0000	0.0000	0.0000	1.0000	1.0000
Type	5,563	2.7235	0.8091	1.0000	3.0000	3.0000	3.0000	4.0000
Num	5,563	0.9891	1.6174	0.0000	0.0000	0.0000	1.7918	5.2575
RHELevel-1	5,563	0.1738	0.3790	0.0000	0.0000	0.0000	0.0000	1.0000
Media	5,563	0.4774	0.4995	0.0000	0.0000	0.0000	1.0000	1.0000
RLE	5,563	0.4859	0.4998	0.0000	0.0000	0.0000	1.0000	1.0000
Size	5,563	22.1288	1.2241	19.7056	21.2799	21.9496	22.8257	26.0209
ROA	5,563	0.0091	0.1204	−0.5965	0.0028	0.0273	0.0634	0.2867
Lev	5,563	0.4390	0.2272	0.0601	0.2646	0.4267	0.5879	1.1949
Growth	5,563	0.0851	0.6015	−0.7734	−0.1546	0.0000	0.1767	4.3830
Top1	5,563	0.2994	0.1352	0.0798	0.1990	0.2786	0.3791	0.6748
Indep	5,563	0.3832	0.0573	0.3125	0.3333	0.3750	0.4286	0.6000
Board	5,563	2.0843	0.1951	1.6094	1.9459	2.1972	2.1972	2.5649
Dual	5,563	0.3408	0.4740	0.0000	0.0000	0.0000	1.0000	1.0000
Age	5,563	10.7643	7.9024	0.0000	4.0000	10.0000	16.0000	27.0000
SOE	5,563	0.2547	0.4357	0.0000	0.0000	0.0000	1.0000	1.0000
Big4	5,563	0.0498	0.2175	0.0000	0.0000	0.0000	0.0000	1.0000
Institution	5,563	0.3775	0.2346	0.0023	0.1831	0.3680	0.5592	0.8996
Analyst	5,563	0.9485	1.1639	0.0000	0.0000	0.0000	1.7918	3.7842
HHI	5,563	0.1460	0.1491	0.0341	0.0691	0.0841	0.1512	0.8580

*This table presents the descriptive statistics of major variables used. The definitions of all variables are presented in Table 1*.

### Research Hypothesis Testing

#### Impact of COVID-19 on the Disclosure of Management's Earnings Forecast

Table 3 presented the results of hypothesis 1. The results show that the regression coefficients of voluntariness of earnings forecasts (Vol) and precision of performance forecasts (Type) are significantly negative at the 1% statistical level for both COVID-19 and RHELevel-1 as explanatory variables. The above results suggest that the higher the degree to which the registry region is affected by COVID-19, the less inclined companies are to issue voluntary management earnings forecasts and the less accurate, which supports research hypothesis 1.

**Table 3:** Impact of COVID-19 on management's earnings forecast disclosure.

**Variable**	**Vol**		**Type**	
	**(1)**	**(2)**	**(3)**	**(4)**
Num	−0.0171^***^		−0.0259^***^	
	(−4.02)		(−2.84)	
RHELevel-1		−0.0617^***^		−0.0980^***^
		(−4.19)		(−3.00)
Size	−0.0326^***^	−0.0323^***^	0.0376^**^	0.0381^**^
	(−4.14)	(−4.10)	(2.36)	(2.38)
ROA	0.3732^***^	0.3750^***^	0.2298	0.2318
	(4.71)	(4.72)	(1.56)	(1.58)
Lev	−0.1364^***^	−0.1237^***^	0.1094	0.1287
	(−3.40)	(−3.08)	(1.32)	(1.56)
Growth	−0.0267^**^	−0.0259^**^	0.0217	0.0229
	(−2.23)	(−2.15)	(0.84)	(0.89)
Top1	0.1357^**^	0.1294^**^	−0.1057	−0.1155
	(2.14)	(2.04)	(−0.86)	(−0.94)
Indep	0.2114	0.2067	−0.2721	−0.2794
	(1.36)	(1.32)	(−0.91)	(−0.94)
Board	−0.0307	−0.0248	−0.1242	−0.1153
	(−0.64)	(−0.52)	(−1.39)	(−1.29)
Dual	−0.0517^***^	−0.0530^***^	0.0037	0.0018
	(−2.98)	(−3.04)	(0.12)	(0.06)
Age	−0.0119^***^	−0.0117^***^	0.0053^**^	0.0055^**^
	(−11.38)	(−11.21)	(2.10)	(2.19)
SOE	0.0038	0.0015	−0.0646	−0.0681
	(0.20)	(0.08)	(−1.51)	(−1.59)
BIG4	−0.0080	−0.0143	−0.0827	−0.0921
	(−0.25)	(−0.45)	(−0.98)	(−1.09)
Institution	0.0170	0.0219	−0.1630^**^	−0.1557^**^
	(0.40)	(0.51)	(−2.19)	(−2.10)
Analyst	0.0222^***^	0.0203^**^	0.0079	0.0050
	(2.78)	(2.55)	(0.52)	(0.32)
HHI	0.0402	0.0421	−0.0222	−0.0201
	(0.52)	(0.54)	(−0.13)	(−0.12)
Constant	0.9733^***^	0.9526^***^	2.3190^***^	2.2884^***^
	(4.67)	(4.57)	(5.43)	(5.35)
Industry	YES	YES	YES	YES
*N*	5,563	5,563	5,563	5,563
Adj.R^2^	0.1169	0.1161	0.0286	0.0283

*This table presents the results of the impact of COVID-19 on management's earnings forecast disclosure. The definitions of all variables are presented in Table 1*.

*, **, and *** *indicate 10, 5, and 1% significance levels, respectively*.

#### Impact of COVID-19 and Media Scrutiny, on the Disclosure of Management's Earnings Forecast

Hypothesis 2 of the study explored the effect of media scrutiny on the relationship between COVID-19 and the disclosure of management earnings forecasts.

Based on whether the number of media reports and online reports against companies is greater than the median, we divide the full sample into a weak media monitoring group (Group = 0) and a strong media monitoring group (Group = 1), respectively, and use model (1) for group testing. Table 4 shows the regression results. Columns (2), (4), (6), and (8) test the strong media regulation group and find that the coefficients for Num and RHELevel-1 are not significant. Namely, when media monitoring is strong, the on-site monitoring restrictions triggered by the COVID-19 do not significantly affect management earnings forecasts. Columns (1), (3), (5), and (7) test for the weak media monitoring group and find that the coefficients on Num and RHELevel-1 are significant at the 1% level and the cross-model coefficient difference test is significant at the 1 or 10% level of significance. It shows that when media monitoring is weak, on-site monitoring restrictions triggered by COVID-19 will significantly affect management earnings forecast disclosure. The above results indicated that media monitoring significantly influenced the relationship between COVID-19 and management earnings forecasts disclosure, which could exert external governance effectiveness to some extent, which supported hypothesis 2.

**Table 4:** Impact of COVID-19 and media scrutiny on management earnings forecast.

**Variable**	**Vol**				**Type**			
	**Group = 0**	**Group = 1**	**Group = 0**	**Group = 1**	**Group = 0**	**Group = 1**	**Group = 0**	**Group = 1**
	**(1)**	**(2)**	**(3)**	**(4)**	**(5)**	**(6)**	**(7)**	**(8)**
Num	−0.0284^***^	−0.0041			−0.0434^***^	−0.0117		
	(−5.45)	(−0.86)			(−4.27)	(−1.25)		
*P*-value of diff. in coef.	0.0006^***^				0.0586^*^			
RHELevel-1			−0.1393^***^	0.0200			−0.2534^***^	0.0539
			(−6.27)	(1.06)			(−5.85)	(1.46)
*P*-value of diff. in coef.			0.0000^***^				0.0000^***^	
Size	−0.0403^***^	−0.0204^**^	−0.0405^***^	−0.0207^**^	0.0135	0.0661^***^	0.0130	0.0652^***^
	(−3.81)	(−2.32)	(−3.84)	(−2.36)	(0.66)	(3.86)	(0.63)	(3.80)
ROA	0.3392^***^	0.3835^***^	0.3291^***^	0.3906^***^	0.2775^*^	0.1081	0.2541	0.1278
	(4.22)	(4.81)	(4.10)	(4.90)	(1.77)	(0.70)	(1.63)	(0.82)
Lev	−0.1735^***^	−0.0571	−0.1526^***^	−0.0553	0.2064^**^	0.0134	0.2396^***^	0.0185
	(−4.04)	(−1.32)	(−3.57)	(−1.28)	(2.47)	(0.16)	(2.88)	(0.22)
Growth	−0.0254^*^	−0.0278^**^	−0.0203	−0.0284^**^	−0.0476^*^	0.0814^***^	−0.0394	0.0798^***^
	(−1.73)	(−2.16)	(−1.39)	(−2.21)	(−1.67)	(3.25)	(−1.38)	(3.19)
Top1	0.2028^***^	0.0595	0.1791^***^	0.0582	0.1152	−0.3104^**^	0.0746	−0.3139^**^
	(2.93)	(0.92)	(2.59)	(0.90)	(0.85)	(−2.46)	(0.55)	(−2.49)
Indep	0.1875	0.2153	0.1930	0.2181	−0.4276	−0.1173	−0.4121	−0.1098
	(1.00)	(1.37)	(1.03)	(1.38)	(−1.16)	(−0.38)	(−1.13)	(−0.36)
Board	0.1022^*^	−0.1492^***^	0.1136^*^	−0.1479^***^	−0.1914^*^	−0.0249	−0.1731	−0.0212
	(1.73)	(−3.10)	(1.93)	(−3.08)	(−1.66)	(−0.27)	(−1.51)	(−0.23)
Dual	−0.0422^**^	−0.0613^***^	−0.0438^**^	−0.0619^***^	−0.0320	0.0407	−0.0347	0.0390
	(−2.30)	(−3.66)	(−2.38)	(−3.70)	(−0.89)	(1.25)	(−0.97)	(1.20)
Age	−0.0132^***^	−0.0082^***^	−0.0130^***^	−0.0081^***^	0.0035	0.0068^***^	0.0038	0.0071^***^
	(−10.48)	(−6.32)	(−10.37)	(−6.26)	(1.42)	(2.68)	(1.54)	(2.78)
SOE	−0.0243	0.0288	−0.0251	0.0274	−0.0897^**^	−0.0385	−0.0907^**^	−0.0424
	(−1.05)	(1.35)	(−1.09)	(1.29)	(−1.99)	(−0.93)	(−2.02)	(−1.03)
BIG4	−0.0007	−0.0353	−0.0221	−0.0377	0.0829	−0.1902^***^	0.0507	−0.1969^***^
	(−0.02)	(−1.06)	(−0.49)	(−1.13)	(0.93)	(−2.92)	(0.57)	(−3.03)
Institution	−0.0351	0.0270	−0.0326	0.0307	−0.1595^*^	−0.2101^**^	−0.1557^*^	−0.1995^**^
	(−0.83)	(0.60)	(−0.77)	(0.69)	(−1.94)	(−2.41)	(−1.90)	(−2.30)
Analyst	0.0109	0.0425^***^	0.0085	0.0422^***^	−0.0065	0.0187	−0.0099	0.0178
	(1.07)	(5.60)	(0.83)	(5.58)	(−0.33)	(1.26)	(−0.50)	(1.21)
HHI	0.0543	−0.0013	0.0683	0.0053	−0.0183	−0.1186	−0.0027	−0.1006
	(0.67)	(−0.02)	(0.84)	(0.07)	(−0.12)	(−0.77)	(−0.02)	(−0.65)
Constant	0.9398^***^	0.8569^***^	0.9231^***^	0.8521^***^	3.1128^***^	1.4408^***^	3.1007^***^	1.4275^***^
	(3.38)	(3.81)	(3.33)	(3.79)	(5.75)	(3.29)	(5.75)	(3.26)
Industry	YES	YES	YES	YES	YES	YES	YES	YES
*N*	2,907	2,656	2,907	2,656	2,907	2,656	2,907	2,656
Adj.R^2^	0.1333	0.0923	0.1362	0.0925	0.0213	0.0608	0.0266	0.0610

*This table presents the results of the moderating effect of media scrutiny and the impact of COVID−19 on management's earnings forecast. We divide the full sample into Group = 1 (or Group = 0) according to whether the number of media reports and online reports against companies is above (or equal to or below) the median. The definitions of all variables are presented in Table 1*.

*, **, and *** *indicate 10, 5, and 1% significance levels, respectively*.

#### Impact of COVID-19 and Law Environment, on the Disclosure of Management's Earnings Forecast

Hypothesis 3 explored the effect of the legal environment on the relationship between the COVID-19 and management earnings forecast. According to whether the registry region of listed companies is located in Guangdong Province, Shanghai, Zhejiang Province, and Jiangsu Province, the full sample is divided into a weak legal environment group (Group = 2) and a strong legal environment group (Group = 3), respectively, and we use model (1) for group testing. The regression results are shown in Table 5. Columns (2), (4), (6), and (8) test Group = 3 and find that the coefficients of Num and RHELevel-1 are not significant, illustrating that when the legal environment is strong, the on-site monitoring restrictions triggered by COVID-19 do not have a significant impact on management earnings forecast disclosure. Columns (1), (3), (5), and (7) test Group = 2 and find that the coefficients of Num and RHELevel-1 are significant at the 1% level and the cross-model coefficient difference test is also significant at the 1 or 5% level of significance. That is, when the legal environment is weak, the on-site monitoring restrictions triggered by COVID-19 will significantly affect management earnings forecast disclosure. The above results suggest that the legal environment is also capable of exerting better external governance effectiveness, thus influencing the relationship between COVID-19 and management earnings forecast disclosure, which supported hypothesis 3.

**Table 5:** Impact of COVID−19 and law environment on management earnings forecast.

**Variable**	**Vol**				**Type**			
	**Group = 2**	**Group = 3**	**Group = 2**	**Group = 3**	**Group = 2**	**Group = 3**	**Group = 2**	**Group = 3**
	**(1)**	**(2)**	**(3)**	**(4)**	**(5)**	**(6)**	**(7)**	**(8)**
Num	−0.0247^***^	−0.0068			−0.0516^***^	0.0078		
	(−4.75)	(−1.35)			(−5.25)	(0.78)		
*P*-value of diff. in coef.	0.0252^**^				0.0000^***^		
RHELevel-1			−0.1376^***^	0.0236			−0.2131^***^	0.0589
			(−7.48)	(0.95)			(−6.11)	(1.18)
*P*-value of diff. in coef.			0.0000^***^				0.0010^**^	
Size	−0.0547^***^	−0.0079	−0.0555^***^	−0.0071	0.0181	0.0687^***^	0.0160	0.0684^***^
	(−6.23)	(−0.77)	(−6.36)	(−0.69)	(1.09)	(3.33)	(0.97)	(3.32)
ROA	0.3424^***^	0.4202^***^	0.3362^***^	0.4249^***^	0.2375	0.1813	0.2411^*^	0.1818
	(4.45)	(4.98)	(4.40)	(5.04)	(1.64)	(1.07)	(1.67)	(1.07)
Lev	−0.0890^**^	−0.1908^***^	−0.0732^*^	−0.1889^***^	0.0434	0.1934^**^	0.0742	0.1837^*^
	(−2.18)	(−4.08)	(−1.81)	(−4.05)	(0.56)	(2.06)	(0.97)	(1.96)
Growth	−0.0153	−0.0467^***^	−0.0120	−0.0463^***^	0.0297	0.0274	0.0350	0.0277
	(−1.18)	(−3.17)	(−0.92)	(−3.14)	(1.21)	(0.93)	(1.43)	(0.94)
Top1	0.1964^***^	0.1323^**^	0.1954^***^	0.1318^**^	−0.2296^*^	0.0964	−0.2313^*^	0.1001
	(2.88)	(1.99)	(2.88)	(1.98)	(−1.78)	(0.72)	(−1.80)	(0.75)
Indep	0.2709^*^	0.0827	0.2812^*^	0.0775	−0.4361	−0.2168	−0.4163	−0.2007
	(1.66)	(0.44)	(1.73)	(0.42)	(−1.42)	(−0.58)	(−1.35)	(−0.54)
Board	−0.0219	−0.0827	−0.0147	−0.0816	−0.1179	−0.1861	−0.0995	−0.1861
	(−0.43)	(−1.44)	(−0.29)	(−1.42)	(−1.24)	(−1.61)	(−1.05)	(−1.61)
Dual	−0.0013	−0.0859^***^	−0.0032	−0.0857^***^	−0.0080	0.0252	−0.0123	0.0252
	(−0.07)	(−5.03)	(−0.17)	(−5.02)	(−0.23)	(0.73)	(−0.36)	(0.74)
Age	−0.0130^***^	−0.0121^***^	−0.0127^***^	−0.0122^***^	0.0049^**^	0.0028	0.0056^**^	0.0028
	(−10.79)	(−8.92)	(−10.65)	(−9.00)	(2.18)	(1.03)	(2.50)	(1.03)
SOE	0.0245	−0.0424	0.0221	−0.0447^*^	−0.0463	−0.1596^***^	−0.0524	−0.1556^***^
	(1.20)	(−1.62)	(1.09)	(−1.71)	(−1.21)	(−3.04)	(−1.37)	(−2.97)
BIG4	0.0067	−0.0378	0.0145	−0.0444	−0.0398	−0.1442^*^	−0.0287	−0.1395^*^
	(0.17)	(−0.96)	(0.38)	(−1.13)	(−0.55)	(−1.82)	(−0.39)	(−1.77)
Institution	−0.0435	0.0570	−0.0454	0.0592	−0.0901	−0.2397^***^	−0.0884	−0.2423^***^
	(−0.97)	(1.36)	(−1.02)	(1.42)	(−1.06)	(−2.85)	(−1.05)	(−2.89)
Analyst	0.0417^***^	0.0016	0.0382^***^	0.0014	0.0284^*^	−0.0115	0.0207	−0.0111
	(4.88)	(0.19)	(4.52)	(0.16)	(1.76)	(−0.65)	(1.29)	(−0.63)
HHI	0.1842^**^	−0.0430	0.1514^*^	−0.0312	0.0967	−0.1104	0.0497	−0.1184
	(2.22)	(−0.54)	(1.83)	(−0.40)	(0.62)	(−0.70)	(0.32)	(−0.75)
Constant	1.2871^***^	0.8665^***^	1.3071^***^	0.8333^***^	2.7520^***^	1.9008^***^	2.7753^***^	1.9084^***^
	(5.73)	(2.84)	(5.85)	(2.74)	(6.49)	(3.11)	(6.56)	(3.12)
Industry	YES	YES	YES	YES	YES	YES	YES	YES
*N*	2,860	2,703	2,860	2,703	2,860	2,703	2,860	2,703
Adj.R^2^	0.1544	0.1036	0.1642	0.1033	0.0342	0.0362	0.0375	0.0365

*This table presents the results of the moderating effect of the law environment and the impact of COVID−19 on management's earnings forecast. We divide the full sample into Group = 3 (or Group = 2) according to whether the registry region of listed companies is located in Guangdong Province, Shanghai, Zhejiang Province, and Jiangsu Province. The definitions of all variables are presented in Table 1*.

*, **, and *** *indicate 10, 5, and 1% significance levels, respectively*.

## Mechanism Test

The previous tests concluded that under the influence of COVID-19, directors were unable to attend the company's meetings and discussions, while analysts and institutional investors had difficulties in conducting field research, which would lead to a decrease in the on-site supervision ability of the monitoring bodies and thus affect the disclosure of management earnings forecasts. Therefore, we attempt to test the specific mechanism by which the COVID-19 affects management earnings forecasts. Specifically, we use the percentage of board meetings conducted in the form of online meetings between the balance sheet date and the date of management earnings announcement to measure the effect of the COVID-19 epidemic on the directors' on-site supervision. In addition, the number of visits by institutional investors and analysts to listed companies for on-site research at that time is used to measure the impact of COVID-19 on the on-site monitoring by institutional investors and analysts. We use the decline in passenger traffic where the listed company is located in the month when the management issued the profit forecast as a proxy indicator to measure “traffic decline”. Following Judd and Kenny (1981) and Baron and Kenny (1986), the mediating test was carried out in three steps, Path a, Path b, and Path c.

In the implementation of the mediation test, we first examine the relationship between the impact of COVID-19 and the earnings forecast of management by Path a. If α1 is statistically significant then proceed to the next step. Secondly, we examine the relationship between the influence of COVID-19 on the proportion of communication meetings (C-Meeting), whether to conduct field research (Research), and traffic decline through Path b. If β1 is statistically significant, proceed to the next step. If β1 was statistically significant, we proceeded to the next step. Finally, we analyze the mediating effect by Path c. The mediating variables C-Meeting and Research are included in the model. If the absolute value of λ1 is smaller relative to α1 and λ1 is no longer significant, then the full mediating effect is proved, and if λ1 is still significant only the absolute value decreases, then the partial mediating effect is proved.


(2) $$\begin{array}{l} \text{Path a}:\;EarningsForecast\;=\;\alpha_{0}\;+\;\alpha_{1}Num/RHELevel\;-\;1\\ +\;\alpha_{2}Controls\;+\;IND\;+\;\varepsilon \end{array}$$

(3) $$\begin{array}{l} \text{Path b}:\;Mediator\;=\beta_{0}+\beta_{1}Num/RHELevel-1\\ +\beta_{2}Controls+IND+\varepsilon \end{array}$$

(4) $$\begin{array}{l} \text{Path c}:\;EarningsForecast=\lambda_{0}+\lambda_{1}Num/RHELevel-1\\ +\lambda_{2}Mediator+\lambda_{3}Controls\\ +IND+\varepsilon \end{array}$$

### Evidence of the Impact of Directors' On-Site Supervision Restrictions

We tested whether COVID-19 could affect the managements earnings forecasts disclosure by making directors unable to attend meetings of companies to discuss and monitor the situation. The regression results are presented in Tables 6, 7, and the Path a result was described previously and will not be repeated here. In Path b, Num and RHELevel-1 were significantly and positively correlated with correspondence meetings (C-Meeting) at the 1% statistical level. It shows that the more strongly the registry region is affected by COVID-19, the more the listed companies tend to conduct their board meetings online when the directors are unable to attend the company to participate in the discussion and supervision. The coefficients of Num (RHELevel-1) in Path c were −0.0161 and −0.0245 (−0.0598 and −0.0954), also significant at the 1% level, but the absolute values were smaller than those in Path a, indicating that the partial mediating effect is proved. These results illustrate that the COVID-19 was able to affect the disclosure of management's earnings forecasts by making it impossible for directors to attend the company to attend meetings for discussion and supervision.

**Table 6:** Evidence of the impact of directors' on-site supervision restrictions (X = Num).

**Variable**	**Path a**		**Path b**	**Path c**	
	**Vol**	**Type**	**C-Meeting**	**Vol**	**Type**
Num	−0.0171^***^	−0.0259^***^	0.0159^***^	−0.0161^***^	−0.0245^***^
	(−4.02)	(−2.84)	(4.10)	(−3.78)	(−2.67)
C-Meeting				−0.0638^***^	−0.0851^**^
				(−3.60)	(−2.55)
Size	−0.0326^***^	0.0376^**^	0.0196^**^	−0.0314^***^	0.0392^**^
	(−4.14)	(2.36)	(2.33)	(−3.98)	(2.47)
ROA	0.3732^***^	0.2298	−0.1780^**^	0.3618^***^	0.2147
	(4.71)	(1.56)	(−2.33)	(4.54)	(1.45)
Lev	−0.1364^***^	0.1094	0.2225^***^	−0.1222^***^	0.1283
	(−3.40)	(1.32)	(5.55)	(−3.04)	(1.55)
Growth	−0.0267^**^	0.0217	0.0125	−0.0259^**^	0.0227
	(−2.23)	(0.84)	(1.04)	(−2.14)	(0.88)
Top1	0.1357^**^	−0.1057	−0.1613^***^	0.1254^**^	−0.1194
	(2.14)	(−0.86)	(−2.89)	(1.99)	(−0.97)
Indep	0.2114	−0.2721	−0.0664	0.2072	−0.2777
	(1.36)	(−0.91)	(−0.44)	(1.34)	(−0.94)
Board	−0.0307	−0.1242	−0.0249	−0.0323	−0.1263
	(−0.64)	(−1.39)	(−0.53)	(−0.68)	(−1.42)
Dual	−0.0517^***^	0.0037	0.0143	−0.0508^***^	0.0049
	(−2.98)	(0.12)	(0.91)	(−2.93)	(0.15)
Age	−0.0119^***^	0.0053^**^	0.0073^***^	−0.0114^***^	0.0059^**^
	(−11.38)	(2.10)	(6.59)	(−10.94)	(2.34)
SOE	0.0038	−0.0646	−0.0242	0.0023	−0.0667
	(0.20)	(−1.51)	(−1.29)	(0.12)	(−1.57)
BIG4	−0.0080	−0.0827	0.0138	−0.0071	−0.0815
	(−0.25)	(−0.98)	(0.47)	(−0.22)	(−0.97)
Institution	0.0170	−0.1630^**^	−0.0393	0.0145	−0.1664^**^
	(0.40)	(−2.19)	(−1.09)	(0.34)	(−2.23)
Analyst	0.0222^***^	0.0079	0.0079	0.0227^***^	0.0086
	(2.78)	(0.52)	(1.10)	(2.85)	(0.56)
HHI	0.0402	−0.0222	0.0676	0.0445	−0.0164
	(0.52)	(−0.13)	(0.86)	(0.57)	(−0.10)
Constant	0.9733^***^	2.3190^***^	−0.0146	0.9723^***^	2.3177^***^
	(4.67)	(5.43)	(−0.07)	(4.67)	(5.46)
Industry	YES	YES	YES	YES	YES
*N*	5,563	5,563	5,563	5,563	5,563
Adj.R^2^	0.1169	0.0286	0.0763	0.1199	0.0301

*This table presents the results of the mediating effect of directors' on-site supervision restrictions and the impact of the COVID−19 (measured using Num) on management's earnings forecast, including three paths. We use the percentage of correspondence board meetings (C-Meeting) to measure the effect of COVID−19 on the directors' on-site supervision. The definitions of all variables are presented in Table 1*.

*, **, and *** *indicate 10, 5, and 1% significance levels, respectively*.

**Table 7:** Evidence of the impact of directors' on-site supervision restrictions (X = RHELevel-1).

**Variable**	**Path a**		**Path b**	**Path c**	
	**Vol**	**Type**	**C-Meeting**	**Vol**	**Type**
RHELevel-1	−0.0617^***^	−0.0980^***^	0.0285^**^	−0.0598^***^	−0.0954^***^
	(−4.19)	(−3.00)	(2.09)	(−4.05)	(−2.91)
C-Meeting				−0.0664^***^	−0.0889^***^
				(−3.76)	(−2.68)
Size	−0.0323^***^	0.0381^**^	0.0195^**^	−0.0311^***^	0.0398^**^
	(−4.10)	(2.38)	(2.30)	(−3.93)	(2.50)
ROA	0.3750^***^	0.2318	−0.1852^**^	0.3627^***^	0.2153
	(4.72)	(1.58)	(−2.43)	(4.54)	(1.46)
Lev	−0.1237^***^	0.1287	0.2120^***^	−0.1097^***^	0.1475^*^
	(−3.08)	(1.56)	(5.28)	(−2.73)	(1.78)
Growth	−0.0259^**^	0.0229	0.0121	−0.0251^**^	0.0240
	(−2.15)	(0.89)	(1.01)	(−2.06)	(0.93)
Top1	0.1294^**^	−0.1155	−0.1566^***^	0.1190^*^	−0.1294
	(2.04)	(−0.94)	(−2.80)	(1.89)	(−1.05)
Indep	0.2067	−0.2794	−0.0622	0.2025	−0.2849
	(1.32)	(−0.94)	(−0.41)	(1.30)	(−0.96)
Board	−0.0248	−0.1153	−0.0305	−0.0269	−0.1180
	(−0.52)	(−1.29)	(−0.64)	(−0.56)	(−1.32)
Dual	−0.0530^***^	0.0018	0.0154	−0.0519^***^	0.0031
	(−3.04)	(0.06)	(0.98)	(−2.99)	(0.10)
Age	−0.0117^***^	0.0055^**^	0.0071^***^	−0.0113^***^	0.0061^**^
	(−11.21)	(2.19)	(6.46)	(−10.77)	(2.43)
SOE	0.0015	−0.0681	−0.0218	0.0001	−0.0700
	(0.08)	(−1.59)	(−1.16)	(0.00)	(−1.64)
BIG4	−0.0143	−0.0921	0.0208	−0.0129	−0.0903
	(−0.45)	(−1.09)	(0.71)	(−0.40)	(−1.07)
Institution	0.0219	−0.1557^**^	−0.0448	0.0190	−0.1596^**^
	(0.51)	(−2.10)	(−1.24)	(0.45)	(−2.15)
Analyst	0.0203^**^	0.0050	0.0097	0.0209^***^	0.0058
	(2.55)	(0.32)	(1.35)	(2.63)	(0.38)
HHI	0.0421	−0.0201	0.0607	0.0461	−0.0147
	(0.54)	(−0.12)	(0.77)	(0.59)	(−0.09)
Constant	0.9526^***^	2.2884^***^	0.0092	0.9532^***^	2.2892^***^
	(4.57)	(5.35)	(0.04)	(4.59)	(5.38)
Industry	YES	YES	YES	YES	YES
*N*	5,563	5,563	5,563	5,563	5,563
Adj.R^2^	0.1161	0.0283	0.0731	0.1193	0.0298

*This table presents the results of the mediating effect of directors' on-site supervision restrictions and the impact of the COVID-19 (measured using RHELevel-1) on management's earnings forecast, including three paths. We use the percentage of correspondence board meetings (C-Meeting) to measure the effect of COVID-19 on the directors' on-site supervision. The definitions of all variables are presented in Table 1*.

*, **, and *** *indicate 10, 5, and 1% significance levels, respectively*.

### Evidence of the Impact of On-Site Monitoring Restrictions by Institutional Investors and Analysts

We tested whether COVID-19 can affect the management's earnings forecast disclosure by making it difficult for institutional investors and analysts to conduct field research. The regression results are presented in Tables 8, 9, also the Path a result as described previously and will not be repeated here. As shown in Path b, the coefficients on Num and RHELevel-1 are negative and significant at the 1% level, indicating that the more strongly the registry region is affected by COVID-19, the less institutional investors and analysts visit public companies for field research. The coefficients of Num (RHELevel-1) in Path c were −0.0167 and −0.0252 (−0.0592 and −0.0934), also significant at the 1% level, but the absolute values were smaller than those in Path a, indicating that a partial mediating effect exists. The results suggest that COVID-19 can affect management's earnings forecast disclosure by making it difficult for institutional investors and analysts to conduct field research.

**Table 8:** Evidence on the impact of on-site monitoring restrictions by institutional investors and analysts (X = Num).

**Variable**	**Path a**		**Path b**	**Path c**	
	**Vol**	**Type**	**Research**	**Vol**	**Type**
Num	−0.0171^***^	−0.0259^***^	−0.6511^***^	−0.0167^***^	−0.0252^***^
	(−4.02)	(−2.84)	(−2.58)	(−3.93)	(−2.76)
Research				0.0006^**^	0.0011^***^
				(2.07)	(3.09)
Size	−0.0326^***^	0.0376^**^	0.8307	−0.0331^***^	0.0367^**^
	(−4.14)	(2.36)	(1.50)	(−4.19)	(2.30)
ROA	0.3732^***^	0.2298	4.7281	0.3703^***^	0.2247
	(4.71)	(1.56)	(1.21)	(4.68)	(1.53)
Lev	−0.1364^***^	0.1094	1.4544	−0.1372^***^	0.1078
	(−3.40)	(1.32)	(0.75)	(−3.42)	(1.31)
Growth	−0.0267^**^	0.0217	0.0568	−0.0267^**^	0.0216
	(−2.23)	(0.84)	(0.11)	(−2.24)	(0.84)
Top1	0.1357^**^	−0.1057	−3.8159	0.1380^**^	−0.1015
	(2.14)	(−0.86)	(−0.86)	(2.18)	(−0.83)
Indep	0.2114	−0.2721	−9.1945	0.2170	−0.2621
	(1.36)	(−0.91)	(−0.98)	(1.40)	(−0.88)
Board	−0.0307	−0.1242	−3.3599	−0.0287	−0.1206
	(−0.64)	(−1.39)	(−1.05)	(−0.60)	(−1.35)
Dual	−0.0517^***^	0.0037	−0.1519	−0.0516^***^	0.0039
	(−2.98)	(0.12)	(−0.12)	(−2.97)	(0.12)
Age	−0.0119^***^	0.0053^**^	0.0623	−0.0119^***^	0.0052^**^
	(−11.38)	(2.10)	(0.96)	(−11.42)	(2.08)
SOE	0.0038	−0.0646	−1.8045	0.0049	−0.0627
	(0.20)	(−1.51)	(−1.32)	(0.25)	(−1.47)
BIG4	−0.0080	−0.0827	−5.3006	−0.0048	−0.0769
	(−0.25)	(−0.98)	(−1.58)	(−0.15)	(−0.92)
Institution	0.0170	−0.1630^**^	−0.1561	0.0171	−0.1629^**^
	(0.40)	(−2.19)	(−0.05)	(0.40)	(−2.19)
Analyst	0.0222^***^	0.0079	7.6697^***^	0.0176^**^	−0.0004
	(2.78)	(0.52)	(9.80)	(2.14)	(−0.02)
HHI	0.0402	−0.0222	0.7848	0.0397	−0.0230
	(0.52)	(−0.13)	(0.19)	(0.51)	(−0.14)
Constant	0.9733^***^	2.3190^***^	−12.0151	0.9805^***^	2.3320^***^
	(4.67)	(5.43)	(−0.89)	(4.70)	(5.47)
Industry	YES	YES	YES	YES	YES
*N*	5,563	5,563	5,563	5,563	5,563
Adj.R^2^	0.1169	0.0286	0.1220	0.1181	0.0297

*This table presents the results of the mediating effect of on-site monitoring restrictions by institutional investors and analysts and the impact of the COVID-19 (measured using Num) on management's earnings forecast, including three paths. We use the number of visits by institutional investors and analysts to companies for on-site research (Research) to measure the effect of COVID-19 on their on-site monitoring. The definitions of all variables are presented in Table 1*.

*, **, and *** *indicate 10, 5, and 1% significance levels, respectively*.

**Table 9:** Evidence on the impact of on-site monitoring restrictions by institutional investors and analysts (X = RHELevel-1).

**Variable**	**Path a**		**Path b**	**Path c**	
	**Vol**	**Type**	**Research**	**Vol**	**Type**
RHELevel-1	−0.0617^***^	−0.0980^***^	−4.2673^***^	−0.0592^***^	−0.0934^***^
	(−4.19)	(−3.00)	(−4.82)	(−4.03)	(−2.85)
Research				0.0006^**^	0.0011^***^
				(2.02)	(3.01)
Size	−0.0323^***^	0.0381^**^	0.8504	−0.0328^***^	0.0372^**^
	(−4.10)	(2.38)	(1.54)	(−4.15)	(2.33)
ROA	0.3750^***^	0.2318	4.4372	0.3724^***^	0.2271
	(4.72)	(1.58)	(1.12)	(4.69)	(1.55)
Lev	−0.1237^***^	0.1287	2.0217	−0.1249^***^	0.1266
	(−3.08)	(1.56)	(1.03)	(−3.12)	(1.54)
Growth	−0.0259^**^	0.0229	0.1088	−0.0260^**^	0.0228
	(−2.15)	(0.89)	(0.22)	(−2.16)	(0.88)
Top1	0.1294^**^	−0.1155	−4.1375	0.1318^**^	−0.1111
	(2.04)	(−0.94)	(−0.93)	(2.08)	(−0.91)
Indep	0.2067	−0.2794	−9.3903	0.2122	−0.2694
	(1.32)	(−0.94)	(−1.00)	(1.36)	(−0.90)
Board	−0.0248	−0.1153	−3.1399	−0.0230	−0.1119
	(−0.52)	(−1.29)	(−0.99)	(−0.48)	(−1.25)
Dual	−0.0530^***^	0.0018	−0.2039	−0.0528^***^	0.0020
	(−3.04)	(0.06)	(−0.16)	(−3.03)	(0.06)
Age	−0.0117^***^	0.0055^**^	0.0687	−0.0118^***^	0.0054^**^
	(−11.21)	(2.19)	(1.07)	(−11.26)	(2.16)
SOE	0.0015	−0.0681	−1.8732	0.0026	−0.0661
	(0.08)	(−1.59)	(−1.38)	(0.14)	(−1.54)
BIG4	−0.0143	−0.0921	−5.4719	−0.0111	−0.0863
	(−0.45)	(−1.09)	(−1.64)	(−0.35)	(−1.02)
Institution	0.0219	−0.1557^**^	−0.0228	0.0220	−0.1556^**^
	(0.51)	(−2.10)	(−0.01)	(0.52)	(−2.10)
Analyst	0.0203^**^	0.0050	7.5952^***^	0.0158^*^	−0.0031
	(2.55)	(0.32)	(9.79)	(1.93)	(−0.19)
HHI	0.0421	−0.0201	0.5169	0.0418	−0.0207
	(0.54)	(−0.12)	(0.12)	(0.53)	(−0.13)
Constant	0.9526^***^	2.2884^***^	−12.4923	0.9599^***^	2.3016^***^
	(4.57)	(5.35)	(−0.93)	(4.61)	(5.39)
Industry	YES	YES	YES	YES	YES
*N*	5,563	5,563	5,563	5,563	5,563
Adj.R^2^	0.1161	0.0283	0.1240	0.1172	0.0293

*This table presents the results of the mediating effect of on-site monitoring restrictions by institutional investors and analysts and the impact of the COVID-19 (measured using RHELevel-1) on management's earnings forecast, including three paths. We use the number of visits by institutional investors and analysts to companies for on-site research (Research) to measure the effect of the COVID-19 on their on-site monitoring. The definitions of all variables are presented in Table 1*.

*, **, and *** *indicate 10, 5, and 1% significance levels, respectively*.

### Evidence of the Impact of Traffic Decline

We tested whether COVID-19 can affect the management's earnings forecast disclosure by traffic decline. The regression results are presented in Tables 10, 11, also the Path a result was described previously and will not be repeated here. As shown in Path b, the coefficients on Num and RHELevel-1 are positive and significant at the 1% level, indicating that the more strongly the registry region is affected by COVID-19, the more traffic declines. The coefficients of Num (RHELevel-1) in Path c were −0.0162 and −0.0230 (−0.0555 and −0.0796), significant at the 5% level, and the absolute values were smaller than those in Path a, indicating that a partial mediating effect exists. The results suggest that COVID-19 can affect the management's earnings forecast disclosure through traffic decline.

**Table 10:** Evidence of the impact of traffic decline (X = Num).

**Variable**	**Path a**		**Path b**	**Path c**	
	**Vol**	**Type**	**Traffic-decline**	**Vol**	**Type**
	**(1)**	**(2)**	**(3)**	**(4)**	**(5)**
Num	−0.0171^***^	−0.0259^***^	0.0151^***^	−0.0162^***^	−0.0230^**^
	(−4.02)	(−2.84)	(3.57)	(−3.84)	(−2.54)
Traffic-decline				−0.0580^***^	−0.1865^***^
				(−4.69)	(−6.30)
Size	−0.0326^***^	0.0376^**^	−0.0137	−0.0334^***^	0.0350^**^
	(−4.14)	(2.36)	(−1.63)	(−4.23)	(2.21)
ROA	0.3732^***^	0.2298	−0.0942	0.3677^***^	0.2122
	(4.71)	(1.56)	(−1.11)	(4.67)	(1.44)
Lev	−0.1364^***^	0.1094	−0.0274	−0.1379^***^	0.1043
	(−3.40)	(1.32)	(−0.67)	(−3.44)	(1.27)
Growth	−0.0267^**^	0.0217	0.0036	−0.0265^**^	0.0224
	(−2.23)	(0.84)	(0.20)	(−2.13)	(0.90)
Top1	0.1357^**^	−0.1057	−0.0077	0.1353^**^	−0.1071
	(2.14)	(−0.86)	(−0.13)	(2.14)	(−0.88)
Indep	0.2114	−0.2721	0.1703	0.2213	−0.2403
	(1.36)	(−0.91)	(1.11)	(1.42)	(−0.81)
Board	−0.0307	−0.1242	0.1160^**^	−0.0240	−0.1025
	(−0.64)	(−1.39)	(2.26)	(−0.50)	(−1.16)
Dual	−0.0517^***^	0.0037	−0.0171	−0.0527^***^	0.0005
	(−2.98)	(0.12)	(−1.06)	(−3.03)	(0.02)
Age	−0.0119^***^	0.0053^**^	0.0022^*^	−0.0118^***^	0.0057^**^
	(−11.38)	(2.10)	(1.71)	(−11.22)	(2.28)
SOE	0.0038	−0.0646	0.0214	0.0051	−0.0606
	(0.20)	(−1.51)	(0.96)	(0.26)	(−1.42)
BIG4	−0.0080	−0.0827	−0.0107	−0.0086	−0.0847
	(−0.25)	(−0.98)	(−0.32)	(−0.27)	(−1.02)
Institution	0.0170	−0.1630^**^	−0.0046	0.0167	−0.1639^**^
	(0.40)	(−2.19)	(−0.13)	(0.39)	(−2.21)
Analyst	0.0222^***^	0.0079	0.0128	0.0230^***^	0.0103
	(2.78)	(0.52)	(1.56)	(2.87)	(0.67)
HHI	0.0402	−0.0222	−0.1978^***^	0.0287	−0.0591
	(0.52)	(−0.13)	(−3.41)	(0.37)	(−0.36)
Constant	0.9733^***^	2.3190^***^	0.6159^***^	−0.0162^***^	−0.0230^**^
	(4.67)	(5.43)	(2.67)	(−3.84)	(−2.54)
Industry effect	YES	YES	YES	YES	YES
*N*	5,563	5,563	5,563	5,563	5,563
Adj.R^2^	0.1169	0.0286	0.0082	0.1205	0.0397

*This table presents the results of the mediating effect of traffic decline s and the impact of COVID-19 (measured using Num) on management's earnings forecast, including three paths. We use the decline in passenger traffic as a proxy indicator to measure Traffic-decline. The definitions of other variables are presented in Table 1*.

*, **, and *** *indicate 10, 5, and 1% significance levels, respectively*.

**Table 11:** Evidence of the impact of traffic decline (X = RHELevel-1).

**Variable**	**Path a**		**Path b**	**Path c**	
	**Vol**	**Type**	**Traffic-decline**	**Vol**	**Type**
	**(1)**	**(2)**	**(3)**	**(4)**	**(5)**
RHELevel-1	−0.0617^***^	−0.0980^***^	0.1011^***^	−0.0555^***^	−0.0796^**^
	(−4.19)	(−3.00)	(3.03)	(−3.77)	(−2.47)
Traffic-decline				−0.0571^***^	−0.1851^***^
				(−4.53)	(−6.17)
Size	−0.0323^***^	0.0381^**^	−0.0142^*^	−0.0332^***^	0.0354^**^
	(−4.10)	(2.38)	(−1.68)	(−4.19)	(2.24)
ROA	0.3750^***^	0.2318	−0.0871	0.3702^***^	0.2156
	(4.72)	(1.58)	(−1.04)	(4.69)	(1.46)
Lev	−0.1237^***^	0.1287	−0.0406	−0.1261^***^	0.1212
	(−3.08)	(1.56)	(−1.00)	(−3.14)	(1.47)
Growth	−0.0259^**^	0.0229	0.0024	−0.0258^**^	0.0234
	(−2.15)	(0.89)	(0.14)	(−2.07)	(0.94)
Top1	0.1294^**^	−0.1155	−0.0001	0.1294^**^	−0.1155
	(2.04)	(−0.94)	(−0.00)	(2.04)	(−0.95)
Indep	0.2067	−0.2794	0.1750	0.2166	−0.2471
	(1.32)	(−0.94)	(1.13)	(1.38)	(−0.84)
Board	−0.0248	−0.1153	0.1109^**^	−0.0185	−0.0947
	(−0.52)	(−1.29)	(2.18)	(−0.38)	(−1.07)
Dual	−0.0530^***^	0.0018	−0.0159	−0.0538^***^	−0.0011
	(−3.04)	(0.06)	(−0.99)	(−3.09)	(−0.04)
Age	−0.0117^***^	0.0055^**^	0.0020	−0.0116^***^	0.0059^**^
	(−11.21)	(2.19)	(1.60)	(−11.06)	(2.35)
SOE	0.0015	−0.0681	0.0230	0.0029	−0.0638
	(0.08)	(−1.59)	(1.04)	(0.15)	(−1.49)
BIG4	−0.0143	−0.0921	−0.0069	−0.0147	−0.0933
	(−0.45)	(−1.09)	(−0.20)	(−0.46)	(−1.12)
Institution	0.0219	−0.1557^**^	−0.0075	0.0215	−0.1572^**^
	(0.51)	(−2.10)	(−0.21)	(0.50)	(−2.13)
Analyst	0.0203^**^	0.0050	0.0146^*^	0.0211^***^	0.0076
	(2.55)	(0.32)	(1.80)	(2.65)	(0.50)
HHI	0.0421	−0.0201	−0.1912^***^	0.0313	−0.0556
	(0.54)	(−0.12)	(−3.38)	(0.40)	(−0.34)
Constant	0.9526^***^	2.2884^***^	0.6265^***^	0.9884^***^	2.4046^***^
	(4.57)	(5.35)	(2.74)	(4.74)	(5.69)
Industry effect	YES	YES	YES	YES	YES
*N*	5,563	5,563	5,563	5,563	5,563
Adj.R^2^	0.1161	0.0283	0.0124	0.1195	0.0391

*This table presents the results of the mediating effect of traffic decline s and the impact of COVID-19(measured using RHELevel-1) on management's earnings forecast, including three paths. We use the decline in passenger traffic as a proxy indicator to measure Traffic-decline. The definitions of other variables are presented in Table 1*.

*, **, and *** *indicate 10, 5, and 1% significance levels, respectively*.

## Robustness Test

### COVID-19 Impact and Earnings Manipulation

We measure management's manipulation using dominant accrual earnings. Specifically, this article selects the absolute value of discretionary accruals estimated by the modified Jones model affected by performance as a measure. It is calculated as follows:

According to the modified Jones model (Dechow et al., 1995), α1, α2, and α3 are estimated, respectively, by using the OLS method for model (5) by year and industry, and then the estimated coefficients are substituted into the model (6) to calculate non-discretionary accruals, and finally, the non-discretionary accruals estimated according to the model (6) are substituted into the model (7) to obtain the discretionary accruals (DA).


(5) $$\begin{array}{l} TA_{t}/A_{t-1}\;=\;\alpha_{1}\left(1/A_{t-1}\right)\;+\;\alpha_{2}\left(\Delta REV_{t}/A_{t-1}\right)\\ +\alpha_{3}\left(PPE_{t}/A_{t-1}\right)\;+\;\varepsilon_{t} \end{array}$$

(6) $$\begin{array}{l} NDA_{t}\;=\;\alpha_{1}\left(1/A_{t-1}\right)\;+\;\alpha_{2}\left(\Delta REV_{t}/A_{t-1}\;-\;\Delta REC_{t}/A_{t-1}\right)\\ +\alpha_{3}\left(PPE_{t}/A_{t-1}\right)\;+\;\varepsilon_{t} \end{array}$$

(7) $$\begin{array}{l} DA_{t}\;=\;TA_{t}/A_{t-1}\;-\;NDA_{t} \end{array}$$

where TA_t_ is the total accruals, which is equal to the operating profit (NT_t_) in period t minus the net cash flow from operating activities in period t (CFO_t_). A_t−1_ is the total assets from the previous year (t-1). ΔREV_t_ is the change in revenues from the fiscal year. PPE_t_ is the gross value of property, plant, and equipment. NDA_t_ is the non- discretionary accrual of period t adjusted by total assets at the end of period t-1. ΔREC_t_ is the change in account receivables from the preceding year. DA_t_ is the amount of discretionary accrual in period t.

Table 12 presents the results of COVID-19 impact and earnings manipulation. The results show that the regression coefficients of the absolute value of discretionary accruals (ABSDA) are significantly positive at the 1% statistical level for both Num and RHELevel-1 as explanatory variables. The above results suggest that the higher the degree to which the registry region is affected by the COVID-19, the companies are more likely to manipulate earnings. The above results are consistent with the main test results.

**Table 12:** COVID-19 impact on earnings manipulation.

**Variable**	**ABSDA**	
	**(1)**	**(2)**
Num	0.0039^***^	
	(3.32)	
RHELevel-1		0.0107^***^
		(3.10)
Size	−0.0137^***^	−0.0137^***^
	(−5.88)	(−5.91)
ROA	−0.2381^***^	−0.2392^***^
	(−8.47)	(−8.49)
Lev	0.0162	0.0135
	(1.36)	(1.13)
Growth	0.0107^***^	0.0106^***^
	(2.97)	(2.93)
Top1	−0.0507^***^	−0.0494^***^
	(−3.45)	(−3.38)
Indep	0.0507	0.0518
	(1.24)	(1.27)
Board	−0.0074	−0.0088
	(−0.59)	(−0.70)
Dual	0.0056	0.0059
	(1.36)	(1.43)
Age	−0.0010^***^	−0.0011^***^
	(−3.32)	(−3.43)
SOE	−0.0171^***^	−0.0165^***^
	(−3.43)	(−3.33)
BIG4	0.0264^***^	0.0280^***^
	(3.21)	(3.38)
Institution	0.0501^***^	0.0488^***^
	(5.11)	(4.98)
Analyst	−0.0041^**^	−0.0037^*^
	(−2.20)	(−1.95)
HHI	0.0017	0.0007
	(0.11)	(0.04)
Constant	0.3777^***^	0.3830^***^
	(6.45)	(6.53)
Industry Effect	YES	YES
*N*	5,563	5,563
Adj.R^2^	0.2092	0.2070

*This table presents the results of the impact of COVID-19 on the management's earnings manipulation. We use a modified Jones model to obtain the discretionary accruals (DA) and take the absolute value (ABSDA) as the explained variable. The definitions of other variables are presented in Table 1*.

*, **, and *** *indicate 10, 5, and 1% significance levels, respectively*.

### COVID-19 Impact and Forecast Accuracy

Referring to the research of Krishnan et al. (2012), we take the deviation of the earnings forecast value in the performance forecast from the actual value as a measure of the forecast accuracy and use it as a sensitivity measure of the dependent variable. The specific calculation formula is as follows:


(8) $$\begin{array}{l} Bias\;=\;|\frac{Forecast\text{\_}Netprofit\;-\;Actual\text{\_}Netprofit}{Marketvalue}|\;*\;100 \end{array}$$

Forecast_Netproft represents the estimated net profit disclosed in the performance forecast. The net profit disclosed in the form of a point estimate is the estimated value, and the net profit disclosed in the form of a closed interval is the average of the upper and lower bounds of the interval. Actual_Netproft is the actual net profit during the forecast period, and Marketvalue is the market value of the stock. When the bias value is larger, it means that the forecast accuracy is lower.

Table 13 presents the results of COVID-19 impact and forecast accuracy. The results show that the regression coefficients of bias are significantly positive at the 1% statistical level for both Num and RHELevel-1 as explanatory variables. The above results suggest that the higher the degree to which the registry region is affected by COVID-19, the lower the forecast accuracy. The above results are consistent with the main test results.

**Table 13:** COVID-19 impact on forecast accuracy.

**Variable**	**Bias**	
	**(1)**	**(2)**
Num	0.2702^***^	
	(5.42)	
RHELevel-1		1.2487^***^
		(6.07)
Size	−0.1451^*^	−0.1509^*^
	(−1.74)	(−1.79)
ROA	−1.3704	−1.3488
	(−1.10)	(−1.08)
Lev	2.3740^***^	2.1621^***^
	(4.46)	(4.06)
Growth	0.1530	0.1375
	(0.63)	(0.57)
Top1	0.0735	0.1855
	(0.12)	(0.29)
Indep	1.6248	1.7039
	(1.10)	(1.14)
Board	0.0600	−0.0335
	(0.13)	(−0.07)
Dual	0.2288	0.2488
	(1.32)	(1.43)
Age	0.0300^**^	0.0275^**^
	(2.55)	(2.33)
SOE	−0.5991^***^	−0.5659^***^
	(−2.92)	(−2.73)
BIG4	−0.0225	0.0670
	(−0.07)	(0.22)
Institution	0.3146	0.2454
	(0.83)	(0.64)
Analyst	−0.2411^***^	−0.2099^***^
	(−3.58)	(−3.08)
HHI	−0.0601	−0.0423
	(−0.10)	(−0.07)
Constant	3.0359	3.3171
	(1.43)	(1.56)
Industry Effect	YES	YES
*N*	5,563	5,563
Adj.R^2^	0.0480	0.0504

*This table presents the results of the impact of COVID-19 on the management's earnings forecast accuracy. Referring to the research of Gopal et al., we take the deviation of the earnings forecast value in the performance forecast from the actual value (Bias) as a measure of the forecast accuracy. When the bias value is larger, it means that the forecast accuracy is lower. The definitions of other variables are presented in Table 1*.

*, **, and *** *indicate 10, 5, and 1% significance levels, respectively*.

### Variable Sensitivity Test

To make the results more robust, we carried out a sensitivity test on the explanatory variable. We re-measured the explanatory variable using the cumulative number of confirmed diagnoses in the listed company locality (Num-Confirmed) and whether it was a secondary response state (RHELevel-2).

Table 14 shows that the regression coefficients of voluntariness of earnings forecasts (Vol) and precision of performance forecasts (Type) are significantly negative at the 1% statistical level for both Num-Confirmed and RHELevel-2 as explanatory variables.

**Table 14:** Impact of COVID-19 on the management's earnings forecast disclosure.

**Variable**	**Vol**		**Type**	
	**(1)**	**(2)**	**(3)**	**(4)**
Num-confirmed	−0.0107^***^		−0.0245^***^	
	(−3.10)		(−3.50)	
RHELevel-2		−0.0497^***^		−0.1221^***^
		(−3.96)		(−4.84)
Size	−0.0325^***^	−0.0324^***^	0.0379^**^	0.0382^**^
	(−4.12)	(−4.09)	(2.38)	(2.40)
ROA	0.3758^***^	0.3745^***^	0.2254	0.2204
	(4.71)	(4.72)	(1.51)	(1.50)
Lev	−0.1345^***^	−0.1282^***^	0.1061	0.1201
	(−3.34)	(−3.19)	(1.28)	(1.46)
Growth	−0.0255^**^	−0.0265^**^	0.0244	0.0222
	(−2.09)	(−2.21)	(0.96)	(0.87)
Top1	0.1322^**^	0.1324^**^	−0.1108	−0.1103
	(2.09)	(2.09)	(−0.90)	(−0.90)
Indep	0.2067	0.1948	−0.2796	−0.3088
	(1.33)	(1.25)	(−0.94)	(−1.04)
Board	−0.0266	−0.0314	−0.1193	−0.1315
	(−0.55)	(−0.65)	(−1.33)	(−1.47)
Dual	−0.0510^***^	−0.0530^***^	0.0061	0.0017
	(−2.93)	(−3.04)	(0.19)	(0.05)
Age	−0.0118^***^	−0.0118^***^	0.0053^**^	0.0053^**^
	(−11.29)	(−11.25)	(2.11)	(2.14)
SOE	0.0070	0.0026	−0.0552	−0.0650
	(0.36)	(0.13)	(−1.28)	(−1.53)
BIG4	−0.0139	−0.0116	−0.0895	−0.0834
	(−0.43)	(−0.36)	(−1.06)	(−0.99)
Institution	0.0201	0.0171	−0.1611^**^	−0.1691^**^
	(0.47)	(0.40)	(−2.16)	(−2.28)
Analyst	0.0221^***^	0.0206^***^	0.0090	0.0058
	(2.76)	(2.59)	(0.58)	(0.38)
HHI	0.0378	0.0399	−0.0376	−0.0349
	(0.48)	(0.51)	(−0.23)	(−0.21)
Constant	0.9916^***^	0.9797^***^	2.3845^***^	2.3638^***^
	(4.76)	(4.70)	(5.51)	(5.55)
Industry Effect	YES	YES	YES	YES
*N*	5,563	5,563	5,563	5,563
Adj.R^2^	0.1157	0.1161	0.0299	0.0311

*This table presents the results of the impact of COVID-19 on the management's earnings forecast disclosure. We re-measured the explanatory variable using the cumulative number of confirmed diagnoses in the listed company locality (Num-Confirmed) and whether it was a secondary response state (RHELevel-2). The definitions of other variables are presented in Table 1*.

*, **, and *** *indicate 10, 5, and 1% significance levels, respectively*.

Table 15 shows that columns (2), (4), (6), and (8) test for the weak media regulation group and find that the coefficients for Num-Confirmed and RHELevel-2 are not significant. Namely, when media monitoring is strong, the on-site monitoring restrictions triggered by the COVID-19 do not significantly affect management earnings forecasts. Columns (1), (3), (5), and (7) test for the weak media monitoring group and find that the coefficients on Num-Confirmed and RHELevel-2 are significant at the 1% level and the cross-model coefficient difference test is significant at the 1% level of significance.

**Table 15:** Impact of COVID-19 and media scrutiny on management earnings forecast.

**Variable**	**Vol**				**Type**			
	**Group = 0**	**Group = 1**	**Group = 0**	**Group = 1**	**Group = 0**	**Group = 1**	**Group = 0**	**Group = 1**
	**(1)**	**(2)**	**(3)**	**(4)**	**(5)**	**(6)**	**(7)**	**(8)**
Num-Confirmed	−0.0181^***^	−0.0045			−0.0407^***^	−0.0088		
	(−4.59)	(−1.17)			(−5.29)	(−1.17)		
*P*-value of diff. in coef.	0.0071^***^				0.0373^**^		
RHELevel-2			−0.0956^***^	0.0079			−0.2429^***^	0.0093
			(−5.47)	(0.50)			(−7.17)	(0.30)
*P*-value of diff. in coef.			0.0000^***^				0.0000^***^	
Size	−0.0400^***^	−0.0199^**^	−0.0396^***^	−0.0206^**^	0.0139	0.0668^***^	0.0149	0.0656^***^
	(−3.78)	(−2.26)	(−3.75)	(−2.34)	(0.68)	(3.89)	(0.73)	(3.83)
ROA	0.3416^***^	0.3820^***^	0.3365^***^	0.3894^***^	0.2705^*^	0.1085	0.2533	0.1213
	(4.25)	(4.79)	(4.19)	(4.89)	(1.73)	(0.70)	(1.63)	(0.78)
Lev	−0.1650^***^	−0.0592	−0.1594^***^	−0.0539	0.2139^**^	0.0114	0.2257^***^	0.0213
	(−3.85)	(−1.36)	(−3.72)	(−1.24)	(2.57)	(0.13)	(2.72)	(0.25)
Growth	−0.0196	−0.0279^**^	−0.0217	−0.0282^**^	−0.0369	0.0809^***^	−0.0415	0.0803^***^
	(−1.33)	(−2.17)	(−1.48)	(−2.20)	(−1.29)	(3.23)	(−1.46)	(3.21)
Top1	0.1934^***^	0.0597	0.1871^***^	0.0582	0.1000	−0.3104^**^	0.0835	−0.3126^**^
	(2.79)	(0.92)	(2.70)	(0.90)	(0.74)	(−2.46)	(0.62)	(−2.48)
Indep	0.1578	0.2173	0.1698	0.2178	−0.4803	−0.1139	−0.4528	−0.1156
	(0.84)	(1.38)	(0.90)	(1.38)	(−1.31)	(−0.37)	(−1.24)	(−0.38)
Board	0.1028^*^	−0.1482^***^	0.1006^*^	−0.1470^***^	−0.1960^*^	−0.0221	−0.2040^*^	−0.0206
	(1.74)	(−3.08)	(1.70)	(−3.06)	(−1.70)	(−0.24)	(−1.78)	(−0.22)
Dual	−0.0418^**^	−0.0606^***^	−0.0430^**^	−0.0618^***^	−0.0307	0.0417	−0.0334	0.0392
	(−2.27)	(−3.61)	(−2.34)	(−3.70)	(−0.86)	(1.28)	(−0.94)	(1.20)
Age	−0.0132^***^	−0.0082^***^	−0.0131^***^	−0.0081^***^	0.0035	0.0069^***^	0.0038	0.0070^***^
	(−10.47)	(−6.31)	(−10.39)	(−6.24)	(1.41)	(2.71)	(1.54)	(2.76)
SOE	−0.0187	0.0308	−0.0216	0.0276	−0.0761^*^	−0.0355	−0.0816^*^	−0.0417
	(−0.81)	(1.44)	(−0.94)	(1.30)	(−1.69)	(−0.85)	(−1.82)	(−1.01)
BIG4	−0.0159	−0.0363	−0.0155	−0.0375	0.0651	−0.1933^***^	0.0686	−0.1951^***^
	(−0.35)	(−1.09)	(−0.34)	(−1.13)	(0.74)	(−2.98)	(0.78)	(−3.00)
Institution	−0.0311	0.0252	−0.0416	0.0313	−0.1525^*^	−0.2106^**^	−0.1788^**^	−0.1995^**^
	(−0.74)	(0.56)	(−0.98)	(0.70)	(−1.86)	(−2.41)	(−2.18)	(−2.29)
Analyst	0.0104	0.0430^***^	0.0085	0.0420^***^	−0.0053	0.0192	−0.0092	0.0173
	(1.02)	(5.65)	(0.83)	(5.55)	(−0.27)	(1.30)	(−0.47)	(1.17)
HHI	0.0452	−0.0011	0.0689	0.0031	−0.0628	−0.1165	−0.0156	−0.1103
	(0.55)	(−0.01)	(0.85)	(0.04)	(−0.40)	(−0.75)	(−0.10)	(−0.71)
Constant	0.9948^***^	0.8581^***^	0.9346^***^	0.8484^***^	3.2809^***^	1.4406^***^	3.1633^***^	1.4260^***^
	(3.56)	(3.82)	(3.37)	(3.77)	(6.05)	(3.29)	(5.88)	(3.25)
Industry effect	YES	YES	YES	YES	YES	YES	YES	YES
*N*	2,907	2,656	2,907	2,656	2,907	2,656	2,907	2,656
Adj.R^2^	0.1308	0.0925	0.1334	0.0922	0.0246	0.0607	0.0324	0.0603

*This table presents the results of the moderating effect of media scrutiny and the impact of COVID-19 on the management's earnings forecast. We re-measured the explanatory variable using the cumulative number of confirmed diagnoses in the listed company locality (Num-Confirmed) and whether it was a secondary response state (RHELevel-2). We divide the full sample into Group = 1 (or Group = 0) according to whether the number of media reports and online reports against companies is above (or equal to or below) the median. The definitions of other variables are presented in Table 1*.

*, **, and *** *indicate 10, 5, and 1% significance levels, respectively*.

Table 16 shows that columns (2), (4), (6), and (8) test for the Group=3 and find that the coefficients of Num-Confirmed and RHELevel-2 are not significant, illustrating that when the legal environment is strong, the on-site monitoring restrictions triggered by the COVID-19 do not have a significant impact on management earnings forecast disclosure. Columns (1), (3), (5), and (7) test for the Group = 2 and find that the coefficients of Num-Confirmed and RHELevel-2 are significant at the 1% level and the cross-model coefficient difference test is also significant at the 1% level of significance. The above results are consistent with the main test results.

**Table 16:** Impact of the COVID-19 and law environment on management earnings forecast.

**Variable**	**Vol**				**Type**			
	**Group = 2**	**Group = 3**	**Group = 2**	**Group = 3**	**Group = 2**	**Group = 3**	**Group = 2**	**Group = 3**
	**(1)**	**(2)**	**(3)**	**(4)**	**(5)**	**(6)**	**(7)**	**(8)**
Num-Confirmed	−0.0237^***^	0.0050			−0.0547^***^	0.0139		
	(−6.54)	(1.16)			(−8.03)	(1.62)		
*P*-value of diff. in coef.	0.0003^***^				0.0000^***^		
RHELevel-2			−0.0964^***^	−0.0056			−0.2741^***^	0.0355
			(−5.83)	(−0.33)			(−8.85)	(1.05)
P-value of diff. in coef.			0.0008^***^				0.0000^***^	
Size	−0.0555^***^	−0.0070	−0.0549^***^	−0.0074	0.0168	0.0688^***^	0.0189	0.0685^***^
	(−6.34)	(−0.68)	(−6.27)	(−0.72)	(1.02)	(3.34)	(1.15)	(3.33)
ROA	0.3321^***^	0.4247^***^	0.3427^***^	0.4222^***^	0.2082	0.1816	0.2199	0.1849
	(4.33)	(5.04)	(4.47)	(5.00)	(1.44)	(1.07)	(1.53)	(1.09)
Lev	−0.0983^**^	−0.1848^***^	−0.0828^**^	−0.1867^***^	0.0192	0.1946^**^	0.0520	0.1881^**^
	(−2.41)	(−3.96)	(−2.04)	(−4.00)	(0.25)	(2.08)	(0.68)	(2.01)
Growth	−0.0119	−0.0468^***^	−0.0130	−0.0466^***^	0.0374	0.0264	0.0359	0.0281
	(−0.92)	(−3.17)	(−1.00)	(−3.16)	(1.54)	(0.89)	(1.48)	(0.95)
Top1	0.1969^***^	0.1304^*^	0.1993^***^	0.1308^**^	−0.2285^*^	0.0964	−0.2211^*^	0.0988
	(2.89)	(1.96)	(2.93)	(1.96)	(−1.79)	(0.72)	(−1.73)	(0.74)
Indep	0.2515	0.0690	0.2545	0.0733	−0.4825	−0.2235	−0.4884	−0.1985
	(1.55)	(0.37)	(1.56)	(0.39)	(−1.58)	(−0.60)	(−1.60)	(−0.53)
Board	−0.0163	−0.0811	−0.0198	−0.0829	−0.1078	−0.1846	−0.1219	−0.1808
	(−0.32)	(−1.41)	(−0.39)	(−1.44)	(−1.14)	(−1.60)	(−1.29)	(−1.56)
Dual	0.0000	−0.0865^***^	−0.0039	−0.0859^***^	−0.0042	0.0231	−0.0131	0.0255
	(0.00)	(−5.06)	(−0.21)	(−5.02)	(−0.12)	(0.67)	(−0.38)	(0.74)
Age	−0.0129^***^	−0.0122^***^	−0.0128^***^	−0.0122^***^	0.0048^**^	0.0028	0.0050^**^	0.0028
	(−10.84)	(−9.01)	(−10.71)	(−8.96)	(2.16)	(1.02)	(2.22)	(1.03)
SOE	0.0352^*^	−0.0474^*^	0.0232	−0.0448^*^	−0.0204	−0.1630^***^	−0.0464	−0.1583^***^
	(1.73)	(−1.81)	(1.15)	(−1.71)	(−0.53)	(−3.10)	(−1.22)	(−3.02)
BIG4	0.0038	−0.0470	0.0106	−0.0427	−0.0459	−0.1468^*^	−0.0271	−0.1431^*^
	(0.10)	(−1.19)	(0.27)	(−1.09)	(−0.63)	(−1.86)	(−0.37)	(−1.81)
Institution	−0.0466	0.0603	−0.0457	0.0584	−0.0992	−0.2392^***^	−0.1027	−0.2371^***^
	(−1.04)	(1.44)	(−1.02)	(1.39)	(−1.18)	(−2.85)	(−1.22)	(−2.82)
Analyst	0.0428^***^	0.0008	0.0383^***^	0.0014	0.0318^**^	−0.0128	0.0219	−0.0114
	(5.03)	(0.09)	(4.52)	(0.15)	(1.99)	(−0.72)	(1.38)	(−0.65)
HHI	0.1728^**^	−0.0222	0.1561^*^	−0.0338	0.0690	−0.0927	0.0116	−0.1157
	(2.09)	(−0.28)	(1.88)	(−0.43)	(0.44)	(−0.58)	(0.07)	(−0.73)
Constant	1.3828^***^	0.8198^***^	1.3208^***^	0.8492^***^	2.9762^***^	1.8680^***^	2.8585^***^	1.8815^***^
	(6.16)	(2.69)	(5.89)	(2.78)	(7.05)	(3.05)	(6.80)	(3.07)
Industry effect	YES	YES	YES	YES	YES	YES	YES	YES
*N*	2,860	2,703	2,860	2,703	2,860	2,703	2,860	2,703
Adj.R^2^	0.1603	0.1034	0.1577	0.1030	0.0466	0.0369	0.0511	0.0364

*This table presents the results of the moderating effect of the law environment and the impact of the COVID-19 on management's earnings forecast. We re-measured the explanatory variable using the cumulative number of confirmed diagnoses in the listed company locality (Num-Confirmed) and whether it was a secondary response state (RHELevel-2). We divide the full sample into Group = 3 (or Group = 2) according to whether the registry regions of listed companies are located in Guangdong Province, Shanghai, Zhejiang Province, and Jiangsu Province. The definitions of other variables are presented in Table 1*.

*, **, and *** *indicate 10, 5, and 1% significance levels, respectively*.

### An Alternative Measure of Media

It is the negative news that tends to have a monitoring effect, positive, and neutral news does not have the effect. In view of this, referring to the research of Core et al. (2008), this article uses negative media reports as a proxy variable for media monitoring. The measurement is as follows: by reading the news about each listed company for the year 2020 provided by the in-depth data of the WIND database one by one. The news for each year is divided into three groups: positive, negative, and neutral. The criteria for the grouping are based on whether the news is favorable to the company's stock price. If the news is theoretically favorable to the company's stock price, it is positive news; otherwise, it is negative information; if the news may have no impact on the stock price or if the nature of the news cannot be judged, it is neutral news.

First, it is grouped by whether the number of the negative news is greater than the median. If the number of negative news against listed companies is greater than the median, the value is 1 (Group = 1), otherwise, it is 0 (Group = 0). Table 17 shows the regression results. Columns (2), (4), (6), and (8) test for the strong media monitoring group and find that the coefficients for Num and RHELevel-1 are not significant or less significant than the Group = 0 under the same conditions. Namely, when media monitoring is strong, the on-site monitoring restrictions triggered by the COVID-19 do not significantly affect management earnings forecasts. Columns (1), (3), (5), and (7) test for the weak media monitoring group and find that the coefficients for Num and RHELevel-1 are significant at the 1% level and the cross-model coefficient difference test is significant at the 1 or 5% level of significance. It shows that when media monitoring is weak, on-site monitoring restrictions triggered by the COVID-19 will significantly affect management earnings forecast disclosure.

**Table 17:** Impact of COVID-19 and Media Scrutiny on the management earnings forecast.

**Variable**	**Vol**				**Type**			
	**Group = 0**	**Group = 1**	**Group = 0**	**Group = 1**	**Group = 0**	**Group = 1**	**Group = 0**	**Group = 1**
	**(1)**	**(2)**	**(3)**	**(4)**	**(5)**	**(6)**	**(7)**	**(8)**
Num	−0.0227^***^	−0.0077			−0.0511^***^	−0.0022		
	(−4.32)	(−1.60)			(−4.94)	(−0.24)		
*P*-value of diff. in coef.	0.0142^**^				0.0426^**^		
RHELevel-1			−0.1171^***^	0.0007			−0.2885^***^	0.0773^**^
			(−5.30)	(0.03)			(−6.65)	(2.12)
*P*-value of diff. in coef.			0.0000^***^				0.0000^***^	
Size	−0.0340^***^	−0.0272^***^	−0.0341^***^	−0.0273^***^	0.0166	0.0479^***^	0.0161	0.0469^***^
	(−3.25)	(−3.16)	(−3.27)	(−3.17)	(0.81)	(2.90)	(0.79)	(2.84)
ROA	0.3262^***^	0.3841^***^	0.3134^***^	0.3906^***^	0.5739^***^	−0.1712	0.5389^***^	−0.1572
	(3.74)	(5.18)	(3.60)	(5.27)	(3.35)	(−1.20)	(3.15)	(−1.11)
Lev	−0.1811^***^	−0.0456	−0.1663^***^	−0.0403	0.2693^***^	−0.0228	0.3037^***^	−0.0230
	(−3.99)	(−1.12)	(−3.67)	(−0.99)	(3.02)	(−0.29)	(3.42)	(−0.29)
Growth	−0.0361^**^	−0.0185	−0.0329^**^	−0.0192	−0.0340	0.0587^**^	−0.0267	0.0572^**^
	(−2.39)	(−1.49)	(−2.19)	(−1.54)	(−1.14)	(2.46)	(−0.90)	(2.40)
Top1	0.2241^***^	0.0227	0.2181^***^	0.0208	0.0164	−0.1976	0.0031	−0.1912
	(3.25)	(0.35)	(3.17)	(0.32)	(0.12)	(−1.59)	(0.02)	(−1.54)
Indep	0.1004	0.2680^*^	0.0818	0.2647^*^	−0.3392	−0.2638	−0.3821	−0.2678
	(0.53)	(1.73)	(0.43)	(1.71)	(−0.91)	(−0.89)	(−1.03)	(−0.90)
Board	0.0256	−0.1010^**^	0.0301	−0.0988^**^	−0.2202^*^	−0.0402	−0.2101^*^	−0.0406
	(0.43)	(−2.14)	(0.51)	(−2.09)	(−1.88)	(−0.44)	(−1.80)	(−0.45)
Dual	−0.0668^***^	−0.0376^**^	−0.0664^***^	−0.0389^**^	−0.0426	0.0436	−0.0417	0.0436
	(−3.64)	(−2.25)	(−3.63)	(−2.33)	(−1.18)	(1.36)	(−1.16)	(1.36)
Age	−0.0131^***^	−0.0076^***^	−0.0131^***^	−0.0074^***^	0.0011	0.0109^***^	0.0013	0.0110^***^
	(−10.38)	(−5.82)	(−10.33)	(−5.71)	(0.43)	(4.33)	(0.52)	(4.41)
SOE	−0.0253	0.0280	−0.0229	0.0258	−0.0330	−0.0935^**^	−0.0268	−0.0924^**^
	(−1.11)	(1.30)	(−1.01)	(1.20)	(−0.73)	(−2.26)	(−0.60)	(−2.25)
BIG4	0.0075	−0.0387	−0.0054	−0.0421	0.0180	−0.1392^**^	−0.0102	−0.1437^**^
	(0.18)	(−1.09)	(−0.13)	(−1.18)	(0.22)	(−2.03)	(−0.12)	(−2.10)
Institution	−0.0646	0.0404	−0.0676	0.0463	−0.1592^*^	−0.1910^**^	−0.1667^**^	−0.1890^**^
	(−1.51)	(0.92)	(−1.58)	(1.06)	(−1.90)	(−2.27)	(−1.99)	(−2.25)
Analyst	0.0173^*^	0.0366^***^	0.0157^*^	0.0358^***^	−0.0161	0.0308^**^	−0.0195	0.0317^**^
	(1.87)	(4.62)	(1.70)	(4.52)	(−0.88)	(2.03)	(−1.07)	(2.09)
HHI	0.0609	−0.0486	0.0673	−0.0429	0.0231	−0.1660	0.0349	−0.1407
	(0.80)	(−0.58)	(0.88)	(−0.51)	(0.15)	(−1.03)	(0.23)	(−0.87)
Constant	1.0501^***^	0.8696^***^	1.0590^***^	0.8584^***^	2.9055^***^	1.9420^***^	2.9359^***^	1.9386^***^
	(3.77)	(4.00)	(3.80)	(3.94)	(5.30)	(4.64)	(5.37)	(4.64)
Industry	YES	YES	YES	YES	YES	YES	YES	YES
*N*	2,969	2,594	2,969	2,594	2,969	2,594	2,969	2,594
Adj.R^2^	0.1355	0.0820	0.1382	0.0811	0.0263	0.0563	0.0327	0.0579

*This table presents the results of the moderating effect of media scrutiny and the impact of COVID-19 on the management's earnings forecast. We divide the full sample into Group = 1 (or Group = 0) according to whether the number of negative media reports against listed companies is above (or equal to or below) the median. The definitions of all variables are presented in Table 1*.

*, **, and *** *indicate 10, 5, and 1% significance levels, respectively*.

Second, it is grouped by whether it has negative news. If there is negative news about the listed companies, the value is 1 (Group = 1), otherwise, it is 0 (Group = 0). Table 18 shows the regression results. Columns (2), (4), (6), and (8) test for the strong media monitoring group and find that the coefficients for Num and RHELevel-1 are not significant or less significant, smaller than the Group = 0 under the same conditions. Namely, when media monitoring is strong, the on-site monitoring restrictions triggered by the COVID-19 do not significantly affect management earnings forecasts. Columns (1), (3), (5), and (7) test for the weak media monitoring group and find that the coefficients for Num and RHELevel-1 are significant at the 1% level and the cross-model coefficient difference test is significant at the 1 or 5% level of significance. It shows that when media monitoring is weak, on-site monitoring restrictions triggered by the COVID-19 will significantly affect management earnings forecast disclosure.

**Table 18:** Impact of COVID-19 and media tone on the management earnings forecast.

**Variable**	**Vol**				**Type**			
	**Group = 0**	**Group = 1**	**Group = 0**	**Group = 1**	**Group = 0**	**Group = 1**	**Group = 0**	**Group = 1**
	**(1)**	**(2)**	**(3)**	**(4)**	**(5)**	**(6)**	**(7)**	**(8)**
Num	−0.0275^***^	−0.0113^***^			−0.0935^***^	0.0002		
	(−3.15)	(−2.90)			(−4.98)	(0.02)		
*P*-value of diff. in coef.	0.0342^**^				0.0296^**^		
RHELevel-1			−0.1706^***^	−0.0279^*^			−0.6089^***^	0.0231
			(−4.42)	(−1.78)			(−7.42)	(0.79)
*P-*value of diff. in coef.			0.0011^***^				0.0000^***^	
Size	−0.0763^***^	−0.0297^***^	−0.0727^***^	−0.0296^***^	−0.0579	0.0455^***^	−0.0449	0.0454^***^
	(−3.74)	(−4.29)	(−3.58)	(−4.28)	(−1.32)	(3.51)	(−1.04)	(3.50)
ROA	0.4866^***^	0.3128^***^	0.4592^***^	0.3149^***^	0.7682^**^	0.0674	0.6663^**^	0.0711
	(3.11)	(5.24)	(2.94)	(5.27)	(2.29)	(0.60)	(2.01)	(0.63)
Lev	−0.0830	−0.0854^***^	−0.0815	−0.0775^**^	0.0506	0.1064^*^	0.0533	0.1051^*^
	(−0.92)	(−2.66)	(−0.91)	(−2.41)	(0.26)	(1.76)	(0.28)	(1.74)
Growth	−0.0886^***^	−0.0129	−0.0833^***^	−0.0127	−0.0644	0.0357^*^	−0.0460	0.0353^*^
	(−3.53)	(−1.24)	(−3.33)	(−1.22)	(−1.19)	(1.83)	(−0.86)	(1.81)
Top1	0.3720^***^	0.0471	0.3780^***^	0.0415	0.4854^**^	−0.2788^***^	0.5043^**^	−0.2780^***^
	(3.30)	(0.90)	(3.37)	(0.79)	(2.01)	(−2.84)	(2.11)	(−2.84)
Indep	−0.2657	0.2960^**^	−0.2935	0.2939^**^	−0.6095	−0.1420	−0.6986	−0.1419
	(−0.79)	(2.30)	(−0.88)	(2.28)	(−0.84)	(−0.59)	(−0.98)	(−0.59)
Board	−0.0100	−0.0383	−0.0053	−0.0338	−0.1135	−0.0493	−0.0961	−0.0489
	(−0.10)	(−0.96)	(−0.05)	(−0.84)	(−0.52)	(−0.66)	(−0.45)	(−0.65)
Dual	−0.0702^**^	−0.0541^***^	−0.0647^**^	−0.0557^***^	−0.1074	0.0240	−0.0891	0.0240
	(−2.31)	(−3.97)	(−2.14)	(−4.09)	(−1.64)	(0.94)	(−1.38)	(0.94)
Age	−0.0171^***^	−0.0080^***^	−0.0176^***^	−0.0078^***^	−0.0031	0.0064^***^	−0.0047	0.0064^***^
	(−7.31)	(−7.80)	(−7.58)	(−7.66)	(−0.62)	(3.34)	(−0.95)	(3.35)
SOE	−0.0034	0.0110	0.0031	0.0093	−0.0903	−0.0485	−0.0670	−0.0484
	(−0.07)	(0.67)	(0.07)	(0.57)	(−0.91)	(−1.57)	(−0.68)	(−1.57)
BIG4	−0.0022	−0.0153	−0.0357	−0.0181	0.1749	−0.1458^**^	0.0601	−0.1469^**^
	(−0.04)	(−0.49)	(−0.61)	(−0.58)	(1.37)	(−2.49)	(0.48)	(−2.51)
Institution	−0.0814	−0.0101	−0.0836	−0.0051	−0.1815	−0.0894	−0.1894	−0.0889
	(−1.24)	(−0.29)	(−1.28)	(−0.14)	(−1.29)	(−1.35)	(−1.36)	(−1.35)
Analyst	−0.0194	0.0401^***^	−0.0223	0.0390^***^	−0.0357	0.0195	−0.0451	0.0197
	(−1.24)	(6.11)	(−1.44)	(5.94)	(−1.06)	(1.59)	(−1.37)	(1.60)
HHI	0.1211	0.0209	0.1308	0.0233	0.3266	−0.0757	0.3571	−0.0711
	(0.90)	(0.34)	(0.98)	(0.38)	(1.13)	(−0.65)	(1.25)	(−0.61)
Constant	1.8628^***^	0.8609^***^	1.8304^***^	0.8444^***^	4.1341^***^	1.9667^***^	4.0107^***^	1.9616^***^
	(3.73)	(4.73)	(3.68)	(4.64)	(3.86)	(5.75)	(3.79)	(5.74)
Industry	YES	YES	YES	YES	YES	YES	YES	YES
N	1,118	4,445	1,118	4,445	1,118	4,445	1,118	4,445
Adj.R^2^	0.1918	0.0788	0.1989	0.0777	0.0650	0.0308	0.0899	0.0310

*This table presents the results of the moderating effect of media scrutiny and the impact of the COVID-19 on the management's earnings forecast. We divide the full sample into Group = 1 (or Group = 0) according to whether there is negative news about the listed companies or not. The definitions of all variables are presented in Table 1*.

*, **, and *** *indicate 10, 5, and 1% significance levels, respectively*.

The empirical results in Tables 17, 18 remain consistent with the results of hypothesis 2. The above results show that higher-level of media monitoring activities could significantly reduce the negative impact of COVID-19 on management earnings forecast disclosure.

## Conclusions

The contagious nature and dangers of COVID-19 add more uncertainty to the unfavorable condition of the economic downturn overlaid with the impact of the outbreak, threatening interests of investors, the enhancement of company value, and the smooth operation of the capital market. Based on this, the article takes listed companies in 2020 as the research object. We found that the more they are affected by COVID-19, the less likely listed companies were to issue voluntary management earnings forecasts, and the less accurate the earnings forecasts were. Media monitoring and the legal environment help to mitigate the negative impact of the pandemic on the management's earnings forecast disclosure. The mechanism test indicated that the more the registry region is affected by COVID-19, the more companies tend to conduct their board meetings by correspondence, and it is difficult for institutional investors and analysts to conduct field research. That effect will greatly reduce the ability of independent directors to monitor the site, which in turn affects the disclosure of management earnings forecasts.

The findings of this article are revealing in both regulatory and practice. As for regulatory, the results can help regulators to strengthen the supervision of management earnings disclosure. Our findings reveal that external governance factors including media monitoring and the legal environment could mitigate the negative impact of the COVID-19 epidemic on management earnings forecasts disclosure. Therefore, regulation should further guide the media to effectively monitor the capital market while consolidating and strengthening the regional rule of law environment to affect the critical role of the external governance system. As for practice, it helps stakeholders perceive the impact of managers' opportunistic behaviors and develop reasonable and effective strategies to deal with it accordingly. We find that the limited ability of stakeholders to monitor the company on-site significantly contributes to the managers' opportunistic behavior. Therefore, for companies, board meetings should be carefully chosen. For analysts and institutional investors, there should be more visits to companies to obtain real information that is not easily concealed.

## Data Availability Statement

The original contributions presented in the study are included in the article/supplementary material, further inquiries can be directed to the corresponding author/s.

## Author Contributions

XF and YX: conceptualization, methodology, and writing. FZ: software. LZ: validation. All authors have read and agreed to the published version of the manuscript.

## Funding

This research was supported by the National Natural Science Foundation of China (Grant Nos.: 71572009 and 71872010), Research Project for Young Teachers of Nanjing Audit University (2021QNPY007), and the Priority Academic Program Development of Jiangsu Higher Education Institutions.

## Conflict of Interest

The authors declare that the research was conducted in the absence of any commercial or financial relationships that could be construed as a potential conflict of interest.

## Publisher's Note

All claims expressed in this article are solely those of the authors and do not necessarily represent those of their affiliated organizations, or those of the publisher, the editors and the reviewers. Any product that may be evaluated in this article, or claim that may be made by its manufacturer, is not guaranteed or endorsed by the publisher.
